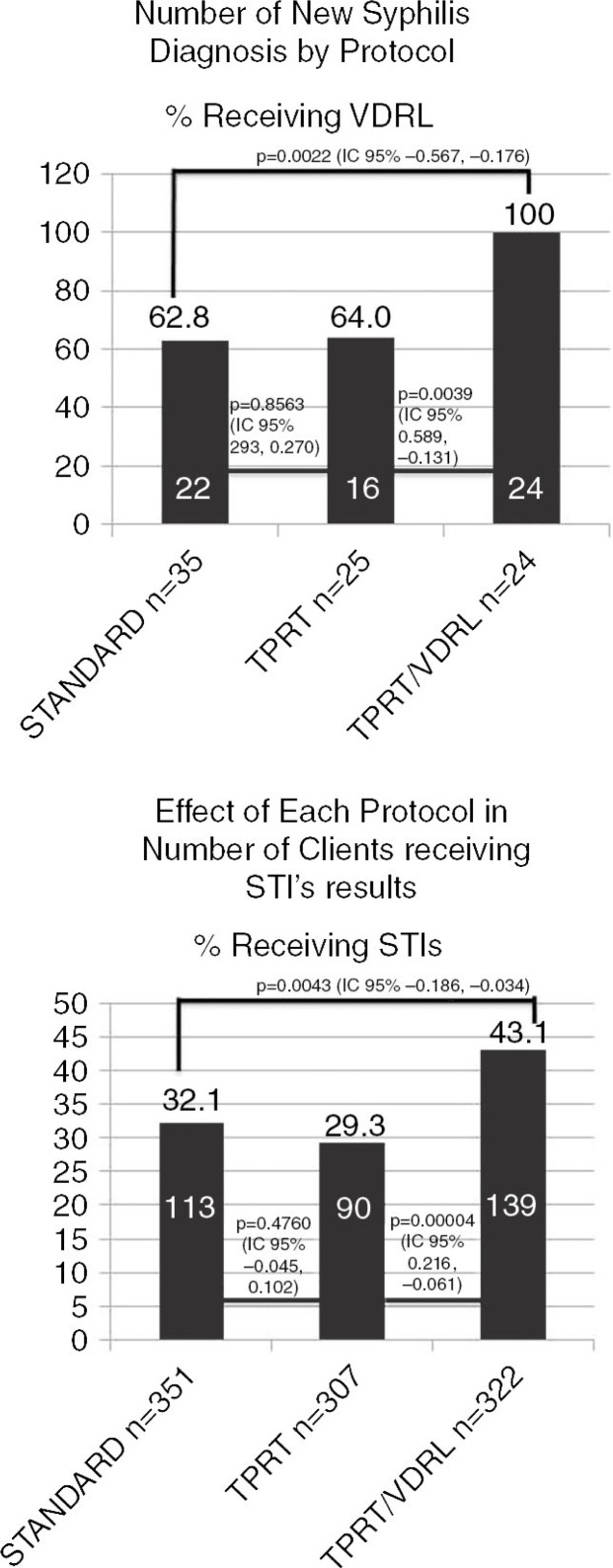# HIV Drug Therapy in the Americas 8–10 May 2014, Rio de Janeiro, Brazil

**DOI:** 10.7448/IAS.17.2.19180

**Published:** 2014-05-07

**Abstract P3–Figure 1:**