# Supplementary material for: HIV Drug Therapy in the Americas 8–10 May 2014, Rio de Janeiro, Brazil
Source: J Int AIDS Soc. 2014 May 7;17(2Suppl 1):19180. doi: 10.7448/IAS.17.2.19180 (PMC4015307; doi:10.7448/IAS.17.2.19180)
Supplement: Related texts

# Sub article: O111: Keynote lecture – Cascade of continuous care in Brazil: present and future challenges

**DOI:** 10.7448/IAS.17.2.19187

**Published:** 2014-05-07

**Authors:** Caldas de Mesquita Fábio, Pascom Ana Roberta Pati, Habckost Dutra de Barros Clarissa, Machado Givisiez Juliana, dos Santos Christ Maria Taques, de Faro Valverde Larissa, Araújo de Freitas Marcelo

**Affiliations:** Department of STI, AIDS and Viral Hepatitis, Ministry of Health of Brazil, Brasilia, Brazil

## Abstract

**Introduction**: By the end of 2013, Brazil has implemented treatment as prevention (TasP) to every person living with HIV (PLWHA) regardless of their CD4 count, aimed at the reduction of HIV transmission. The HIV/AIDS epidemic is unevenly distributed across the territory, being concentrated in capital cities and along the coast.

**Methodology**: The following public information systems were used in this study: Laboratory Tests Control System (SISCEL), Medication Logistics Control System (SICLOM), Notifiable Diseases Information System (SINAN) and Mortality Information System (SIM). Estimations of people who were linked to both public and private HIV health care services were considered.

**Results**: By the end of 2012, 718,000 individuals were living with HIV/AIDS (LWHA) in Brazil and it was estimated that 74% of them were linked to care, of which 313,000 were on ART. Approximately 33% (236,000) of people LWHA had viral load suppression (≤50 copies/ml) and considering those who were on ART, suppression was 76%. Remarkable regional differences in suppression were found. Whilst VL suppression among those who were on ART in the South Region was 77%, in the North Region suppression does not reach 70%.

**Conclusions**: HIV/AIDS epidemic in Brazil follows the standard of its social disparities. The poorer the region, the lower the adherence to treatment and its effectiveness. The implementation of the new strategies in different steps of the cascade as well as the focus on regional hotspots may be the key tools to properly confront the current HIV/AIDS epidemic.

**Abstract O111–Figure 1:**

# Sub article: O112: HIV policy in Central America

**DOI:** 10.7448/IAS.17.2.19188

**Published:** 2014-05-07

**Authors:** Sosa Nestor

**Affiliations:** Gorgas Memorial Institute, Panama City, Panama

## Abstract

There have been significant improvements in the Central America region in HIV policy and care in three areas: (1) access to treatment, (2) reduction of stigma among health care providers and (3) the existence of national strategic plans. But, despite the existence of laws, norms, strategic plans and international agreements to respond to the HIV epidemic in the region, there are important gaps in the implementation, dissemination and monitoring of these policies. Stigma and discrimination against key populations disproportionately affected with HIV is considered a major hurdle in the implementation of HIV policies and still persists in the general population. The lack of political leadership to implement human rights laws for the protection and equality for key populations, and an appropriate response to gender-based violence are two other significant deficiencies that negatively impact the HIV policy environment. A fragmented civil society effort produces lack of cooperation and even competition between the different non-governmental organizations compromising the attainment of common goals. In many countries in the region, a lack of sufficiently strong national authority to solicit resources and coordinate the response has also been identified. In summary, despite advancement in certain areas of HIV policy and care in Central America, there are still many problems and obstacles that need to be addressed in order to improve this region's response to the HIV epidemic.

## References

[1] Kincaid MM, Fortune-Greeley HC, Alvey NW HIV policy assessment in Central America. USAID.

# Sub article: O113: Co-morbidities and ageing in HIV

**DOI:** 10.7448/IAS.17.2.19189

**Published:** 2014-05-07

**Authors:** Reiss Peter

**Affiliations:** Academic Medical Centre, University of Amsterdam, Amsterdam, The Netherlands

## Abstract

Combination antiretroviral therapy (cART) can successfully prevent traditional HIV-associated morbidity and mortality, thereby allowing patients with HIV to survive and become increasingly older. Not surprisingly, ageing-associated co-morbidities are increasingly being observed and importantly influence clinical management and care. The risk of some of these co-morbidities has been demonstrated to be increased in the context of uncontrolled infection, but even those with suppressed viraemia on cART seem to be at increased risk of the development of ageing-associated non-communicable co-morbidities, including cardiovascular, chronic kidney, liver and pulmonary disease, diabetes mellitus, osteoporosis, non-AIDS associated malignancies and neurocognitive impairment. The underlying pathogenesis is multifactorial and, next to traditional (often lifestyle-related) risk factors, seems to include sustained immune activation and inflammation, both systemically and within particular body compartments such as the central nervous system. In addition, circumscript toxicities resulting from both past and present exposure to particular antiretrovirals may also contribute to various co-morbidities such as, for example, chronic kidney disease, osteoporosis and cardiovascular disease. Whether chronic inflammation and cART exposure may also affect and potentially accelerate the biology of ageing and thereby enhance the premature onset of ageing-associated co-morbidities is an area of active investigation.

# Sub article: O121: Keynote lecture – The gaps and successes of TB control in the Americas: the GHESKIO experience in Haiti

**DOI:** 10.7448/IAS.17.2.19190

**Published:** 2014-05-07

**Authors:** Pape Jean William

**Affiliations:** Division of Infectious Diseases, Weill Cornell Medical College, New York, NY, USA and Les Centres GHESKIO, Port-au-Prince, Haiti

## Abstract

Tuberculosis (TB) has declined more rapidly in the Americas than in other regions of the world: from 1990 to 2012, TB prevalence and mortality decreased respectively from 103/100,000 to 40/100,000 and from 5.9 to 1.9/100,000. Similarly the incidence of TB has been reduced from 59 to 29/100,000. In 2012, only 3% of the TB cases worldwide occurred in the Americas. Haiti, Bolivia and Guyana have the highest TB incidence (Haiti 213; Bolivia 127; Guyana 109; per 100,000). Haiti and Bolivia are also the only two countries in the region for which the WHO is unable to estimate TB mortality using vital registration measurements. TB prevalence in Haiti has increased after the 2010 earthquake with 13% more cases nationwide in 2012. TB treatment success rates in the Americas are relatively low, with a 75% cure/completion rate (84% in Haiti) vs. 87% for the world. Outcomes are particularly poor for retreatment cases, with a cure/completion rate of only 62% (72% in Haiti); 10% of retreatment cases died, 4% failed and 24% defaulted. An estimated 2.2% of new cases and 14% of retreatment cases in the Americas have MDR-TB. Yet, in 2012 only 5481 of 23,811 (23%) retreatment cases were tested for MDR-TB. The management of HIV-TB co-infection is also challenging, with 16% of TB patients co-infected with HIV. Only 56% of TB patients have been tested for HIV (81% in Haiti), and of the 20,355 known to be co-infected, only 13,699 (76%) are on antiretroviral therapy, though it is universally recommended. Despite great success in reducing TB burden in the Americas, with much effort to be accomplished by Haiti, Bolivia and Guyana, there are gaps in care that must be bridged for the entire region.

# Sub article: O122: TB prevention and care

**DOI:** 10.7448/IAS.17.2.19191

**Published:** 2014-05-07

**Authors:** Sued Omar

**Affiliations:** Fundación Huésped, Buenos Aires, Argentina

## Abstract

In 2012, the Pan American Health Organization estimated 280,000 new tuberculosis cases in the Americas, with an estimated incidence of 29/100,000 individuals, 16% associated with HIV. Brazil, Peru, Mexico, Haiti, Colombia and Bolivia account for 72% of the cases in the region. In large countries, tuberculosis is concentrated in certain populations, in particular among urban high-risk groups including HIV individuals, immigrants from TB high-incidence countries and underserved populations such as homeless and those with a history of drug and alcohol abuse. At the country level, elimination of TB requires the implementation of multiple measures focused on different layers of the population. The particular complexities of big cities, such as population density and social structure, create specific opportunities for transmission, but also enable targeted TB control interventions. The presentation will describe the current TB situation in the Americas, the most affected populations, and the general and specific social, educational, operational, legal and monitoring TB control interventions required for the control, as well as providing some recommendations with particular emphasis on HIV-related TB. In addition, a summary of new tools for prevention and control, including new technologies, vaccines and new drugs will be presented.

# Sub article: O131: The HIV treatment response prediction system: using the experience of treating tens of thousands of patients to guide optimal drug selection

**DOI:** 10.7448/IAS.17.2.19192

**Published:** 2014-05-07

**Authors:** Revell Andrew, Wang Dechao, Reiss Peter, van Sighem Ard, Hamers Raph, Morrow Carl, Wood Robin, Gazzard Brian, Montaner Julio, Lane H Clifford, Larder Brendan

**Affiliations:** 1 HIV Resistance Response Database Initiative, London, UK; 2 HIV Resistance Response Database Initiative, Bioinformatics, London, UK; 3 Department of Global Health, Academic Medical Centre of the University of Amsterdam, Amsterdam, The Netherlands; 4 Stichting HIV Monitoring, Amsterdam, The Netherlands; 5 Desmond Tutu HIV Centre, University of Cape Town, Cape Town, South Africa; 6 Chelsea and Westminster Hospital, St Stephen's Clinic, London, UK; 7 BC Centre of Excellence in HIV/AIDS, Vancouver, Canada; 8 Division of Clinical Research, National Institute of Allergy and Infectious Diseases, National Institutes of Health, Bethesda, MD, USA

## Abstract

**Introduction**: Optimizing the selection of antiretroviral drugs for patients experiencing virological failure remains a challenge, particularly in resource-limited settings where the latest drugs may not be accessible and genotyping may not be affordable to guide drug selection. The HIV Resistance Response Database Initiative (RDI) was set up in 2002 to develop computational models that predict virological response to different combinations of drugs, and to make those models freely available to assist optimal drug selection.

**Materials and methods**: In order to collect sufficient data to develop such models, HIV cohorts and clinics around the world were approached for a range of longitudinal clinical, virological and treatment data for their patients. These data have been used to train a range of neural network, support vector machine, linear regression and random forest models to predict virological response to combination therapy. The models were tested with independent validation sets and their accuracy compared with that of genotyping with rules-based interpretation.

**Results**: Data from approximately 110,000 patients have been collected. The best performing models (random forests) routinely predict virological response/failure to a change of therapy with an accuracy of 80% or more, even without the use of a genotype. Moreover, they are significantly more accurate than genotyping itself. They are able to identify simple, less costly, alternative drug combinations that are predicted to be effective for the majority of cases that failed following a treatment change in the clinic. The models are used to power the free online HIV Treatment Response Prediction System HIV-TRePS.

**Conclusions**: HIV-TRePS, in essence, makes the distilled experience of hundreds of physicians treating tens of thousands of patients available to all. Use of the system to help guide antiretroviral treatment selection following virological failure has significant potential to improve patient outcomes and save costs following a switch to antiretroviral drug therapy, particularly in resource-limited settings.

# Sub article: O132: STaR: single tablet regimen RPV/FTC/TDF is safe and well tolerated compared to EVF/FTC/TDF in ART-naïve Latinos at week 96

**DOI:** 10.7448/IAS.17.2.19193

**Published:** 2014-05-07

**Authors:** Hawkins Trevor, Cohen Cal, Wohl David, Arribas Jose, Henry Keith, Bloch Mark, Towner William, Garner Will, Porter Danielle, Walker Ivan, Gonzalez Jose Luis, Scharen-Guivel Valeska

**Affiliations:** 1 Infectious Diseases, Southwest Care Center, Santa Fe, NM, USA; 2 Community Research Initiative of New England, HIV/Infectious Diseases, Boston, MA, USA; 3 Infectious Diseases, University of North Carolina, Chapel Hill, NC, USA; 4 Infectious Diseases, Hospital Universitario La Paz, Madrid, Spain; 5 Infectious Diseases, Hennepin County Medical Center, Minneapolis, MN, USA; 6 Infectious Diseases, Holdsworth House Medical Practice, Darlinghurst, Australia; 7 Infectious Diseases, Kaiser Permanente Medical Center, Los Angeles, CA, USA; 8 Gilead Sciences, Biostatistics HIV, Foster City, CA, USA; 9 Clinical Virology, Gilead Sciences, Foster City, CA, USA; 10 Gilead Sciences, MS HIV East, Foster City, CA, USA; 11 Gilead Sciences, Spain HIV Medical, Foster City, CA, USA; 12 Gilead Sciences, Medical Affairs HIV, Foster City, CA, USA

## Abstract

**Introduction**: STaR is the first study to directly compare the safety and efficacy of two once daily single-tablet regimens (STRs), rilpivirine/emtricitabine/tenofovir DF (RPV/FTC/TDF) and efavirenz/emtricitabine/tenofovir DF (EFV/FTC/TDF).

**Materials and methods**: STaR is an open-label, randomized 1:1, 96-week study in treatment-naïve HIV-1-infected subjects. The primary endpoint was the proportion of subjects with HIV-1 RNA <50 copies/ml at Week 48 (12% non-inferiority margin; snapshot analysis). Additional analyses were performed to assess safety and efficacy in Latino subjects.

**Results**: Overall for the primary endpoint, RPV/FTC/TDF (*n*=394) was non-inferior to EFV/FTC/TDF (*n*=392; 86% vs. 82%) by Snapshot analysis for HIV RNA <50 copies/ml (difference 4.1%, 95% CI [−1.1%, 9.2%]) at Week 48 and also at Week 96 (78% vs. 72%; difference 5.5%, 95% CI [−0.6%, 11.5%]). In the Latino subgroup (RPV/FTC/TDF [*n*=59], EFV/FTC/TDF [*n*=75]), the proportion of subjects with HIV-1 RNA <50 copies/ml at Week 48 was 90% in the RPV/FTC/TDF arm vs. 81% in the EFV/FTC/TDF arm (difference 7.9%, 95% CI [−4.3%, 20.1%]) and at Week 96, 83% vs. 75%, respectively (difference 7.8%, 95% CI [−6.3%, 21.9%]). The rates of all treatment-emergent adverse events in >10% of subjects in either arm for the Latino subpopulation by MedDRA Preferred Term for RPV/FTC/TDF and EFV/FTC/TDF (%; respectively) were: abnormal dreams (12%, 27%), anxiety (10%, 8%), bronchitis (12%, 4%), cough (12%, 9%), depression (9%, 13%), diarrhoea (5%, 13%), dizziness (5%, 20%), fatigue (19%, 19%), headache (9%, 12%), insomnia (12%, 19%), nausea (19%; 11%), rash (9%; 16%), sinusitis (12%; 5%), somnolence (2%, 12%) and upper respiratory tract infection (20%, 19%). There were six discontinuations due to adverse events in the Latino population. Of these, one (2%) was in the RPV/FTC/TDF arm and five (7%) were in the EFV/FTC/TDF arm.

**Conclusions**: Treatment-naïve HIV-1-infected Latino subjects had similar virologic success (HIV-1 RNA <50 copies/ml) for RPV/FTC/TDF and EFV/FTC/TDF through 96 weeks. RPV/FTC/TDF demonstrated low rates of adverse events and fewer AE-related discontinuations than EFV/FTC/TDF through Week 96. In the Latino population, RPV/FTC/TDF was safe, well-tolerated and effective.

# Sub article: O133: Clinical outcomes with first-line antiretroviral therapy in Latin America: analysis from the LATINA retrospective cohort

**DOI:** 10.7448/IAS.17.2.19194

**Published:** 2014-05-07

**Authors:** Belloso Waldo, Angriman Federico, Kovalevsky Leandro, Sanchez Jorge, La Rosa Alberto, Rodriguez Loria Gabriela, Alave Jorge, Losso Marcelo

**Affiliations:** 1 Infectious Diseases Unit, Hospital Italiano and CICAL, Buenos Aires, Argentina; 2 Internal Medicine Department, Hospital Italiano and CICAL, Buenos Aires, Argentina; 3 School of Statistics, National University of Rosario, Rosario, Argentina; 4 IMPACTA, Lima, Peru; 5 CICAL, Buenos Aires, Argentina; 6 Hospital JM Ramos Mejia, HIV Unit, Buenos Aires, Argentina

## Abstract

**Introduction**: Most low- to middle-income countries providing antiretroviral therapy for patients in need are located in Latin America. Scarce information about clinical and virological outcomes of this population is, however, available at a regional level. This data could have not only an individual therapeutic impact but also a public health impact. Our objective was to analyze patient characteristics and therapeutic outcomes of newly treated individuals with HIV infection in our region during combination antiretroviral era.

**Materials and methods**: HIV+ adult patients who initiated combination antiretroviral therapy between 2002 and 2006 were selected from the LATINA Retrospective Cohort database, comprising data from five regional reference HIV care centres located in four Latin American countries (Argentina, Brazil, Peru and Mexico). NNRTI and PI-based antiretroviral regimens were compared in terms of main composite outcome of all-cause mortality and AIDS defining clinical events by multivariate logistic regression. Secondary outcomes were non-AIDS clinical events, hazard of change of first-line treatment, time to the first severe event (AIDS defining, non-AIDS defining or death) and time to AIDS by Cox proportional regression models adjusted by site, gender, time since HIV diagnosis, baseline CD4+ T-cell count and AIDS at baseline.

**Results**: A total of 937 patients were included in the analysis. Nearly 25% of them were women. At treatment initiation, patients had a mean age of 37±10 years and a CD4+ T-cell count of 146±118 cells/mm^3^. Three hundred and twenty patients (34%) had AIDS and the median lapse from HIV diagnosis was 1.9 years. In 709 patients (75%), the first selection included an NNRTI - in 551 was efavirenz - while 225 patients received a PI-based regimen and 158 of them included lopinavir. One hundred and forty four patients (15%) presented a reported adverse event during the first year of follow-up. No significant difference was detected in the odds for occurrence of the primary outcome, while CD4+ cell count was the only covariate associated with it. The estimated hazard ratio for time to change (NNRTIs vs. PIs) was 0.58 (95% CI 0.45-0.75, *p*<0.01)

**Comments**: Despite current treatment guidelines, patients with HIV infection start antiretroviral therapy with low CD4+ T-cell count in our region, with a significant proportion of them having already had an AIDS diagnosis. NNRTI-based regimens are largely favoured over PI-based treatments as initial selection in Latin America and while the risk of significant clinical events appears to be similar between both strategies, the chance of a therapy switch at one year of follow-up is significantly higher with PI-based regimens.

# Sub article: O211: Keynote lecture – Progress and challenges in prevention of mother-to-child HIV-1 transmission in the United States

**DOI:** 10.7448/IAS.17.2.19195

**Published:** 2014-05-07

**Authors:** Mofenson Lynne

**Affiliations:** Eunice Kennedy Shriver National Institute of Child Health and Human Development, National Institutes of Health, Bethesda, MD, USA

## Abstract

Twenty years ago, PACTG 076 demonstrated that administration of zidovudine during pregnancy, labour and to the newborn for six weeks reduced perinatal transmission by nearly 70%, providing the first demonstration of “treatment as prevention.” In the United States, a comprehensive national response resulted in rapid implementation of the PACTG 076 regimen, with decreases in perinatal transmission from 25% before 1994 to ~5% within two years. Currently, in well-resourced health systems like the United States, where 98% of women receive prenatal care primarily starting in the first trimester, with implementation of opt-out universal HIV testing of pregnant women, the provision of combination antiretroviral therapy (cART) regimens during pregnancy, with individualized, laboratory-guided management; elective caesarean delivery if HIV RNA is >1000 copies/ml near delivery; infant antiretroviral prophylaxis; and avoidance of breastfeeding, the risk of perinatal transmission has been reduced to about 1%, and elimination of new perinatal infections is now possible. Current United States guidelines for use of antiretroviral drugs for maternal health and reduction of perinatal transmission will be discussed, as well as potential challenges to the elimination of new perinatal infections. Such challenges include continuing new infections in women, late/no prenatal care, lack of identification of HIV status and late or no antiretroviral prophylaxis. Additionally, with the dramatic decline in perinatal infections, there are now thousands of HIV-exposed infants who are now happily uninfected, but who have in utero exposure to multiple drugs with limited data on long-term safety. A major challenge – but critical need and ethical obligation – will be to evaluate potential long-term effects of antiretroviral exposures in uninfected children.

# Sub article: O212: Challenges in the prevention of perinatal HIV infection in Latin America

**DOI:** 10.7448/IAS.17.2.19196

**Published:** 2014-05-07

**Authors:** Bologna Rosa

**Affiliations:** Hospital de Pediatria, Buenos Aires, Argentina

## Abstract

In high-income countries, MTCT transmission has been virtually eliminated through universal HIV testing and counselling, access to effective antiretroviral prophylaxis and treatment, safer delivery practices, family planning and safe use of breast-milk substitute. Scientific and programmatic evidence shows that PMTCT interventions can reduce the risk to less than 5% even in breastfeeding populations. Latin America has a prevalence of 1.4 million HIV cases, with 6300 (3600–9600) children newly infected per year. The estimated global rate of MTCT in 2011 for LA was 14.2% [5.8–18.5] (9.2% without breastfeeding). Key targets for 2015 include reducing the MTCT rate to <2% and having less than 0.3 new infant HIV infections per 1000 live births and less than 0.5 congenital syphilis cases per 1000 live births. Increasing antenatal access to medical care, incorporating educational strategies to increase accurate perception of personal risk and including rapid assays to meet the demand, where HIV testing is the main barrier to PMTCT, are ways to improve counselling and testing. The main challenges are to strengthen health systems, and service delivery models that integrate prenatal care, HIV and sexual and reproductive health, and to promote early initiation of prenatal care and improving the quality of prenatal care. The regional coverage of HIV testing in pregnant women increased from 29% in 2008 to 66% in 2011. Calculated coverage of antiretroviral therapy for HIV-infected pregnant women was 70% in 2011. Many countries in the region are close to reaching the goals of elimination of MTCT of HIV. Early diagnosis and treatment saves children's lives. Expanding the availability of early infant diagnostic testing is a critical need. Primary prevention should be re-enforced. There is a need to identify populations that are still not reached and the reasons why they have no access or are not using available services, and to eliminate the gaps of inequity, especially in young women, aborigines and other women in vulnerable situations.

# Sub article: O213: A view on pregnancy among HIV perinatally infected adolescents

**DOI:** 10.7448/IAS.17.2.19197

**Published:** 2014-05-07

**Authors:** della Negra Marinella

**Affiliations:** Instituto de Infectologia Emílio Ribas, Sao Paulo, Brazil

## Abstract

From the 127 HIV perinatally infected girls aged 14 years or older followed by Instituto de Infectologia Emílio Ribas, 30 became pregnant (23.6%), making a total of 39 pregnancies and 35 babies (four miscarriages). Two adolescents got pregnant three times and four got pregnant twice. The gestational age varied from 32 to 39 weeks (mean 36.8 weeks) with three cases of premature birth. Twenty-six adolescent mothers had a fixed sexual partner from which 24 were aware of the adolescent's HIV status. Two adolescents got pregnant through domestic artificial insemination. Three adolescents mentioned the use of illicit drugs while pregnant. Only 50% of the adolescents showed undetectable viral load results by the end of the pregnancy, while the remaining showed results varying from 260 to 67,127 copies/mm^3^ (mean 20,555 copies/mm^3^). Ritonavir boosted lopinavir was part of the antiretroviral treatment to prevent mother-to-child transmission in 82.6% of the cases and all newborns received oral AZT syrup prophylaxis. Caesarean section was the mode of delivery of choice in 32 pregnancies, while vaginal delivery was applied to three cases. Just one of the newborns was HIV infected. The weight at birth ranged from 1900 to 3280 g (mean 2546 g) and the height at birth varied from 42 to 49 cm (mean 45.5 cm). This group of HIV vertically infected adolescents, despite being educated since childhood concerning their HIV status, the risk of HIV transmission through sexual relationships, the care with their sexual partners and the risk of an undesirable pregnancy, showed a high rate of pregnancies.

# Sub article: O214: Non-infectious diseases in perinatally HIV-infected children and adolescents from Latin America and the Caribbean

**DOI:** 10.7448/IAS.17.2.19198

**Published:** 2014-05-07

**Authors:** Machado Daisy Maria, Krauss Margot, Succi Regina Célia, Cruz Maria Letícia, Gomes Ivete, Freimanis Laura, Scotta Marcelo, Goldani Marcelo, Fonseca Rosana, Hazra Rohan

**Affiliations:** 1 Pediatrics, Universidade Federal de São Paulo, São Paulo, Brazil; 2 WESTAT, Rockville, MD, United States; 3 Pediatrics, Hospital dos Servidores do Estado, Rio de Janeiro, Brazil; 4 Pediatric Infectious Diseases, Hospital Geral de Nova Iguaçu, Niterói, Brazil; 5 Obstetrics, Irmandade da Santa Casa de Misericordia de Porto Alegre, Porto Alegre, Brazil; 6 Pediatric Infectious Diseases, Universidade Federal do Rio Grande do Sul, Porto Alegre, Brazil; 7 Pediatrics, Hospital Femina, Porto Alegre, Brazil; 8 Institute of Child Health and Human Development, Pediatric Adolescent Maternal AIDS Branch, Bethesda, MD, USA

## Abstract

**Introduction**: Opportunistic and other infections have declined since the introduction of HAART; however, non-infectious complications (NI) potentially related to HIV disease and ART remain a concern. This study examines the prevalence and incidence of specific NI categories and evaluates risk factors for the occurrence of NI among children enrolled in the NICHD International Site Development Initiative (NISDI) paediatric study in Latin America and the Caribbean.

**Methods**: Perinatally HIV-infected children at sites in Brazil, Argentina, Mexico, Peru and Jamaica were enrolled in a long-term follow-up study and followed for the occurrence of 12 targeted categories of NI. Cross-sectional and longitudinal analyses were performed to calculate prevalence of NI before enrolment and the incidence rates of NI after enrolment. Covariates included age, CDC clinical classification, viral load, WHO immunologic classification, ARV regimen and height/age at enrolment.

**Results**: 537 (52%) of the 1032 vertically infected children had at least one NI prior to enrolment. The most prevalent disorders prior to enrolment were asthma/allergies (29%), malnutrition (24%) and neurologic disorders (18%). After enrolment, 728 new NI diagnoses were made among 464 (45%) children for an overall incidence of 18 per 100 person-years. The most common disorders per 100 person-years were asthma/allergies 9.4 (95% CI; 8.3–10.5), malnutrition 2.9 (95% CI; 2.3–3.5), neurologic 3.5 (95% CI; 2.8–4.1) and behavioural 2.1 (95% CI; 1.6–2.6). NIs related to cardiac, pancreatic, and renal conditions; encephalopathy; and neoplasms were relatively rare in this cohort, with all occurring at a rate<0.5/100 person-years. Those with NIs were significantly more immunosuppressed (*p*=0.01) and more likely to be Category C by CDC classification at enrolment (*p*=0.02). No difference regarding gender, age, viral load or ARV regimen at enrolment was found.

**Conclusions**: The overall incidence of NI diagnoses in this cohort of perinatally HIV-infected children from Latin America and the Caribbean was 18 per 100 person-years. NIs were more common in those with advanced HIV disease, suggesting that these complications are more likely to be related to HIV than to ART. This study also confirms recent evidence in the literature that asthma/allergies are common among HIV-infected children.

# Sub article: O215: Correlation of access to care, VL and CD4 count with health-related quality of life (HRQoL) in HIV-1-infected women: access supports better outcomes

**DOI:** 10.7448/IAS.17.2.19199

**Published:** 2014-05-07

**Authors:** Baran Robert, Cassetti Isabel, Valdez Ramalho Madruga Jose, Krznaric Ivanka, d'Arminio Monforte Antonella, Mulcahy Fiona, Samarina Anna, Xi He, Zachry Woodie, van Wyk Jean, Martinez Marisol

**Affiliations:** 1 AbbVie, Global Health Economics Outcomes Research, North Chicago, IL, USA; 2 Helios Salud, HIV Programs, Buenos Aires, Argentina; 3 Centro de Referencia e Treinamento, DST/AIDS, Sao Paulo, Brazil; 4 Medical Center for Infectious Diseases (MIB), HIV, Berlin, Germany; 5 Department of Health Sciences, Clinic of Infectious and Tropical Diseases, San Paolo Hospital, University of Milan, Milan, Italy; 6 Department of Infectious Diseases, St James Hospital, Trinity College, Dublin, Ireland; 7 Infectious Diseases, Saint Petersburg HIV Centre, Saint Petersburg, Russian Federation; 8 Infectious Disease Department, Guangzhou 8th People's Hospital, Guangzhou, PR, China; 9 AbbVie, Global Medical Affairs, The Woodlands, TX, USA; 10 Virology, AbbVie, Global Medical Affairs, North Chicago, IL, USA

## Abstract

**Introduction**: Global HIV-1 prevalence estimate is currently 33.4 million and women comprise >50% of those infected. The majority of women may lack regular care and only 25% are virologically suppressed [1]. The ELLA study is a cross-sectional, non-interventional epidemiology study conducted across Latin America (LA), Europe, Canada and Asia that describes barriers to care for HIV-infected women and associations with disease stage, treatment effects and HRQoL.

**Methods**: HIV-infected women eligible for ELLA (≥18 years) completed the self-administered Barrier to Care Scale (BACS) comprising 12 items in four domains (index range 0–12, overall range 1–4, greater=more barriers, overall score ≥2 considered severe) and the ACTG Health Status Assessment questionnaire comprising 21 items assessing nine HRQoL domains (range 0-100, greater=better). Healthcare providers documented medical history, HIV infection/co-morbidities data and clinic attendance. This analysis presents correlations of BACS response, last reported VL and CD4 count with HRQoL outcomes. Spearman rank order was used to test correlations with statistical significance set at *p*<0.05.

**Results**: Enrolment included 1931 women from 30 countries, including 519 in LA. Total population mean age was 40 years (16.9% >50 years), education<12 years was noted for 47.7% of subjects, 36% were unemployed and 82.9% resided in an urban area. HIV was acquired heterosexually in 83.0%. Current ART was reported for 88.2% of subjects; 57.5% had a VL<50 c/ml; mean CD4 was 540.5 c/µl. Mean [SD] BACS index and overall scores were 6.19 [3.47] (*N*=1818) and 2.09 [0.71] (*N*=1922), respectively. Overall BACS score was greater (worse) in LA than Western Europe: 2.2 [0.7] vs. 2.0 [0.7] (*p*<0.0001). “Stigma” was a contributing factor (*p*<0.0001) to score in LA. Lower (better) BACS index and overall scores were both correlated with better HRQoL on each of the nine domains (*p*<0.0001). Lower VL was correlated with better HRQoL for general health, physical, role, social and overall domains (*p*<0.0001) as well as mental (*p*=0.0006), cognitive and vitality domains (*p*<0.04). Only the pain domain was not correlated to VL. Greater CD4 count was also correlated to better HRQoL on every domain (*p*≤0.0002) except pain.

**Conclusions**: In HIV-infected women, discordance between regions in overall BACS score was observed. Reduced barriers to care correlated with better HRQoL, as did lower VL and greater CD4 counts. Better access to care may improve HRQoL outcomes in this population.

## References

[1] Centers for Disease Control (CDC)

# Sub article: O221: The role of ART in preventing HIV infections within MSM populations

**DOI:** 10.7448/IAS.17.2.19200

**Published:** 2014-05-07

**Authors:** Phillips Andrew

**Affiliations:** University College London Medical School, London, UK

## Abstract

HIV epidemics in MSM are expanding in countries of all incomes and in many incidences of new infections is also rising. This talk will consider HIV epidemics in MSM and analyze the current and potential future roles of ART and condom use in limiting new infections. The analyses will be mainly based specifically on the UK epidemic as a typical example of an epidemic in MSM that has not been declining over time. The aim will be to discuss findings from modelling analyses in a manner that is readily understandable to non-modellers. The UK provides a good setting to use as an example due to the fact that there are multiple sources of data on various aspects of the epidemic, including on sexual HIV testing behaviour, as well as detailed data on HIV-infected populations. The link between the “cascade of care” and incidence of new infections will be made clear, which emphasizes the critical role of the HIV treatment and care system in controlling the epidemic. Adoption of effective policies to increase HIV testing rates and a change in ART initiation threshold to initiate ART at diagnosis (should this be supported by results from trials which are currently on-going) could potentially have a very high positive impact on HIV incidence. However, ART adherence and retention and condom-less sex are other key determinants of incidence and these need to be dealt with or the impact of higher testing and earlier ART will be lost.

# Sub article: O222: Pre-exposure prophylaxis

**DOI:** 10.7448/IAS.17.2.19201

**Published:** 2014-05-07

**Authors:** Sanchez Jorge

**Affiliations:** Asociacion Civil IMPACTA Salud y Educacion, Lima, Peru

## Abstract

In the Americas, the HIV epidemic is concentrated among men who have sex with men (MSM) and transgender women. In Latin America these groups continue to be hidden, stigmatized and discriminated against, even in health care facilities. The fight against the HIV epidemic in these populations requires tailored research on prevention strategies, structural changes, community participation and political willingness. After disappointing behavioural, vaccine and community level intervention trials, pre-exposure prophylaxis (PREP) has shown efficacy in preventing HIV acquisition in different populations including MSM (iPrEx study). Originally designed to be carried out in Latin America (LA), the iPrEx study was a multicenter clinical trial implemented in 11 sites from nine countries in four different continents. Finally, 74% of the 2499 participants came from either Brazil or the Andean Region. Despite such evidence, only Brazil has moved into the next steps to continue generating evidence to eventually make an informed decision about the potential role of PREP in LA. Implementation of PREP requires operational research to determine a variety of aspects such as priority target sub-populations, delivery systems, cost-effectiveness and public health impact based on “real-world” uptake and adherence data (including facilitators and barriers). Demonstration projects aimed to address these and other relevant factors are on-going in the US and Brazil. In spite of the proven efficacy of antiretrovirals in preventing HIV transmission, it is widely accepted that not a single strategy will be sufficient to curb the HIV epidemic in MSM. New promising interventions, such as rectal microbicides and HIV vaccines, are being evaluated; however, combined strategies will be needed to reach the goal of an AIDS-free generation. The next step in the HIV prevention research agenda for MSM would need to include the evaluation of acceptability, uptake of and adherence to behavioural, biological and structural components of a combined prevention package.

# Sub article: O311A: Important advances in the last year, including highlights from the 21st Conference on Retroviruses and Opportunistic Infections

**DOI:** 10.7448/IAS.17.2.19211

**Published:** 2014-05-07

**Authors:** Soto-Ramírez Luis

**Affiliations:** Instituto Nacional de Ciencias Medicas y Nutricion Salvador Zubiran, Mexico City, Mexico

## Abstract

This year's CROI meeting had important new data on cure, prevention and resistance. While a cure is not yet on the horizon, a number of advances provide clues about how HIV might be eradicated. At 2013 CROI, the case of a baby that started ART 30 hours after birth was presented. After 18 months, treatment was stopped persisting with undetectable viral load despite being off antiretrovirals. This year an update of the case reported that despite being off treatment for two years, this infant still has undetectable blood viral load and extensive testing has not found HIV in her PBMCs or other reservoirs, suggesting that very early therapy for infants may lead to a “functional cure.” In another cohort study, proviral reservoirs were significantly reduced in perinatally infected patients with virologic control on ART by one year of age vs. patients with later virologic control. Prevention efforts have more interesting options. In female macaques, GSK744 long-acting injections every four weeks provide prophylaxis against vaginal SHIV challenge. The most important presentation on prevention (PARTNER study) demonstrated zero HIV transmissions between serodiscordant couples with HIV-infected partner on suppressive ART, despite on-going condom-less sex, stressing the importance of early start on ART, while some other options as gene therapy with cells that do not express CCR5, that showed promising results are ready for human use. Transmitted resistance is still a problem despite higher virological control in treated individuals. Using enhanced sensitivity mutation-specific polymerase chain reaction assay a 70% higher prevalence of key reverse transcriptase mutations was detected, being higher among youths aged 13–19 years vs. older age groups and lower among Hispanic individuals vs. black or white persons. However, the clinical significance of these mutants needs to be demonstrated. Looking for resistance-associated mutations to integrase strand transfer inhibitors, a retrospective study (2000–2013) showed that are very few 1/1617 integrase sequences evaluated (T66T/I mixture).

# Sub article: O311B: Important advances in the last year, including highlights from the 21st Conference on Retroviruses and Opportunistic Infections

**DOI:** 10.7448/IAS.17.2.19210

**Published:** 2014-05-07

**Authors:** Cahn Pedro

**Affiliations:** Fundación Huésped, Buenos Aires, Argentina

## Abstract

In the last year, the HIV/AIDS research field remained active, and so, several good quality papers were produced and new data were presented at the IAS conference in Kuala Lumpur, the European Conference (EACS) in Brussels and the Retrovirus meeting (CROI) in Boston, among other meetings and conferences. New data provide stronger evidence supporting the initiation of antiretroviral therapy in HIV-1-infected individuals regardless of their CD4 count. Also, treatment as prevention has now more data supporting the evidence for the effectiveness of this strategy, as shown in MSM serodiscordant couples. Morbidity and mortality from non-AIDS-defining illness does not differ from that of the general population if CD4 counts above 500 cells/ml are achieved in HIV-infected people. The new WHO guidelines reflect this situation, by adopting the 500 CD4 threshold for treatment initiation, as well as recommending treatment in several situations, regardless of the CD4 cell count. The aim of achieving a functional cure has received further impulse, provided by reports suggesting that if ARV therapy is started very early during acute infection, functional cure may be achievable in some patients. New drugs, with better tolerability profile, “user friendly” in terms of dosing and with high antiretroviral potency have been approved by regulatory authorities in high-income and some middle-income countries. Last but not least, class-sparing strategies have been tested successfully, challenging the triple-drug paradigm. While the vaccine field has failed to move forward, ARV therapy is now simple, frequently based in fixed-dose combinations and easier to comply with. In this presentation, the state of the art on ARV therapy will be discussed.

# Sub article: O321: Managing drug and alcohol use disorders in patients living with HIV/AIDS

**DOI:** 10.7448/IAS.17.2.19203

**Published:** 2014-05-07

**Authors:** Altice Frederick

**Affiliations:** Clinical and Community Research, Yale University School of Medicine, New Haven, CT, USA

## Abstract

Approximately half of all patients living with HIV/AIDS (PLWHA) have underlying substance use disorders (SUDs), including problematic alcohol and drug use. In addition to the direct negative medical and psychiatric consequences associated with SUDs, they in turn are associated with a number of negative HIV treatment outcomes, including increased HIV risk behaviours, delayed HIV diagnosis, delayed linkage and retention in HIV care, decreased likelihood of being prescribed antiretroviral therapy (ART), decreased ART adherence and decreased likelihood of achieving viral suppression. As a result, PLWHA with underlying SUDs should be targeted using evidence-based interventions and provision of adequate support in order to achieve targets set forth for HIV treatment as prevention (TasP) to be optimized. In this presentation, a review of types of SUDs will be presented along with the negative medical and psychiatric consequences and their impact on HIV treatment outcomes. In addition, practical solutions for screening, brief interventions and referral to treatment (SBIRT) as well as evidence-based interventions that promote improved engagement in the HIV continuum of care will be covered to help guide HIV prevention and treatment strategies.

# Sub article: O322: Epidemiological characteristics of attendants of VCT in the context of a faster and integral diagnosis of HIV/STI in Condesa Clinic, Mexico City

**DOI:** 10.7448/IAS.17.2.19204

**Published:** 2014-05-07

**Authors:** Juarez Luis, Uribe Felipe, Iracheta Patricia, Ruiz Veronica, Medina Yazmin, González Andrea

**Affiliations:** 1 HIV Laboratory, Clinica Condesa, Mexico City, Mexico; 2 Population Studies, Colegio de la Frontera Norte, Piedras Negras, Mexico; 3 Condesa Clinic, Coordination HIV Program, Mexico City, Mexico

## Abstract

**Introduction**: This study describes a faster and integral HIV/AIDS laboratory diagnosis for opportune attention and performs the epidemiological characterization of people who attended Voluntary Counselling and Testing (VCT) at Condesa Clinic (CC). On 2010, the HIV laboratory at VCT initiated a one-stop diagnosis approach. It was scaled-up in 2013 by the addition of rapid CD4 counting results available for post-test counselling by peer counsellors the same day.

**Methods**: From November 2011 to September 2012, we studied 10,485 individuals who requested VCT at CC. We applied a rapid HIV test and established an algorithm to confirm all HIV rapid test positive results. Additionally, each serum was assayed to identify antibodies against syphilis, hepatitis B and hepatitis C. CD4 cell counts and viral load were also obtained among HIV-positive individuals (Figure 1).

**Abstract O322–Figure 1:** HIV/STI diagnosis algorithm presently used in Condesa Clinica, Mexico.

HIV/STI diagnosis algorithm presently used in Condesa Clinica, Mexico.

Late HIV infection diagnosis was defined if CD4 counts were lower than 200 cells. Prevalences of HIV and STIs were estimated and risks of HIV infection and late HIV diagnosis were calculated.

**Results**: Women represented 32.3% of all individuals studied. Global seroprevalences of HIV, Syphilis, HBsAg and Anti-HCV markers were 20.1, 6.0, 1.0 and 1.0, respectively. Men had 5.7, 9.3, 14 and 1.6, times higher seroprevalence of HIV, Syphilis, HBsAg and Anti-HCV infections markers than women (Table 1).

**Abstract O322–Table 1:** Global and stratified by sex prevalence and co-infection percent of HIV *, syphilis**, HBsAg and anti-HCV among VCT attendants consecutively studied at Clínica Condesa in Mexico City, from 11 November 2011 to 28 September 2012

Characteristics	Number	Global prevalence & 95% CI	Number	Women's prevalence & 95% CI	Number	Men's prevalence & 95% CI
Marker						
HIV	10,485	20.1 (19.3–20.8)	3390	4.8 (4.0–5.4)	7095	27.5 (16.4–28.4)
Syphilis	10,447	6.0 (5.5–6.4)	3381	0.9 (0.5–1.2)	7066	8.4 (7.7–9.0)
HBsAg	10,392	1.0 (0.7–1.1)	3372	0.1 (0.02–0.14)	6923	1.4 (1.1–1.6)
Anti-HCV	10,396	1.0 (0.7–1.1)	3372	0.7 (0.4–1.0)	7024	1.1 (0.8–1.3)
Co-infection		Co-infection% & 95% CI		Co-infection% & 95% CI		Co-infection% & 95% CI
HIV & Syphilis	10,421	3.3 (2.9–3.6)	3380	0.2 (0.08–0.42)	7041	4.8 (4.3–5.3)
HIV & HCV	10,371	0.5 (0.3–0.6)	3371	0.08 (0.01–0.2)	7000	0.6 (0.45–0.84)
HIV & HBsAg	10,367	0.7 (0.5–0.8)	3371	0	6996	1.0 (0.81–1.3)

Global and stratified by sex prevalence and co-infection percent of HIV *, syphilis**, HBsAg and anti-HCV among VCT attendants consecutively studied at Clínica Condesa in Mexico City, from 11 November 2011 to 28 September 2012

Among women, HIV infection was related to age and syphilis but among men, age, hepatitis B, hepatitis C and syphilis markers were related to HIV infection. Prevalence of late HIV diagnosis was 31.8%. The risk of late HIV diagnosis was significantly higher among women and increases as age increases. There were eight cases of recent HIV infection in younger men who were negative to HIV Ab tests, positive to HIV Ag/Ab test and showed high viral load.

**Conclusions:** A faster, patient-centred HIV diagnosis resulted in a great increase of HIV detections in CC while facilitating more opportune medical attention. This study also provides useful information resulting from the establishment of faster integral HIV infection diagnosis at CC in the context of a population with high HIV infection magnitude and large gender differences on HIV and STI prevalence as well as risk factors for HIV infection and late HIV diagnosis along the period of study.

# Sub article: O323: Late diagnosis in Mexico and its behaviour in time

**DOI:** 10.7448/IAS.17.2.19205

**Published:** 2014-05-07

**Authors:** Villafuerte Adriana, Uribe Patricia, Magis Carlos

**Affiliations:** 1 Centro Nacional para la Prevencin y Control del VIH y el sida, Direccin de Atencin Integral, Mexico City, Mexico; 2 Centro Nacional para la Prevencin y Control del VIH y el sida, Direccin General, Mexico City, Mexico

## Abstract

**Introduction**: The universal antiretroviral treatment access program was established in Mexico in 2003, through the System of Social Protection in Health. By the end of 2013, 57,073 people were under free antiretroviral therapy in the country. The care process is monitored through the Antiretroviral Administration, Logistic and Monitoring System (SALVAR for its acronym in Spanish). This process begins with detection, continues with the entailment of people to health services and continues steadily with the monitoring. The initial step is early diagnosis, avoiding the occurrence of opportunistic infections and death, as well as preventing new infections. In Mexico, timely detection is still a major challenge.

**Goal**: Describe the occurrence of late diagnostics (<200 CD4) in Mexico from 2008 to 2013, as well as the differences between men and women.

**Methods**: Descriptive study conducted in Mexico with the records of the Antiretroviral Administration, Logistic and Monitoring System, men and women with HIV who enter antiretroviral treatment, in the period 2008–2013 (*n*=33,858).

**Results**: On average, each year of the period under analysis, entered around 5650 people enter treatment and approximately 75% were men. The occurrence of late diagnostics throughout the period was 49.9%, noting variability in each year with a downward trend. From 2008 to 2013, a reduction in the proportion of late diagnostics of 10% was observed. In the analysis by sex, the men showed a decrease of 4.8% from the initial year of analysis to 2013 (2008: 132 CD4 – 2013: 147 CD4) and in the case of women, it was 24.5% (2008: 161 CD4 – 2013: 205 CD4), finding a statistically significant difference (*p*<0.0003) between the two groups.

**Conclusions**: A decrease in the occurrence of late diagnoses in Mexico was observed, which reflects efforts in prevention, yet there is still a gap between early detection and the initiation of antiretroviral therapy. Reducing late diagnosis in women is a reflection of increased access to health services in our country, a result of the generation of inclusive and gender-sensitive public policy. There has been a clear increase in the availability and access to information on women's health, encouraging self-care. Currently, more women demand access to HIV testing and to health services in general.

# Sub article: O331: HIV and hepatitis: what's new?

**DOI:** 10.7448/IAS.17.2.19206

**Published:** 2014-05-07

**Authors:** Nelson Mark

**Affiliations:** Chelsea and Westminster Hospital, London, UK

## Abstract

Hepatitis in the context of HIV infection is rapidly changing. The majority of individuals living with hepatitis B and HIV will be fully suppressed on a regimen including tenofovir and as yet there have been no confirmed reports of resistance developing to this drug in vivo. However, there will be some individuals who are not able to receive tenofovir principally due to renal dysfunction, and other strategies including dose reduction and the use of alternative agents may be required. The major revolution in the field of co-infection has been the development of direct acting antivirals to treat hepatitis C. Both in clinical trials and clinical practice the results in the co-infected mirror, or are even better, than that in the mono-infected population and HIV should not be a barrier to individuals receiving such agents. The first direct acting antivirals boceprevir and telaprevir have already been surpassed in efficacy results within clinical trials, by newer protease inhibitors including faldaprevir and somepravir. The results of the use of the polymerase show potentially even higher rates of treatment success in the HIV population when combined with Interferon and ribavarin. The recent results of the PHOTON study show HIV-infected individuals will also respond to Interferon sparring approaches, with similar success rates as in the monoinfected. Results of studies of combinations of oral DAAs in the monoinfected population have shown efficacy rates approaching 100%, with little drug associated toxicity. The results of the SYNGERY study have suggested that individuals receiving such combinations may only need to receive these drugs for six weeks. However, there will remain barriers; these will include access to medications, the cost of the medications and the increasing reports of re-infection within the HIV population. Although treatment strategies are changing dramatically so will the need for the education of the co-infected population to prevent both initial and re-infection.

# Sub article: O332: HCV treatment access for HIV co-infected patients in Latin America

**DOI:** 10.7448/IAS.17.2.19207

**Published:** 2014-05-07

**Authors:** Sierra-Madero Juan

**Affiliations:** National Institute of Medical Sciences and Nutrition, Mexico City, Mexico

## Abstract

Advances in antiretroviral therapy and increased access have brought enormous benefits to affected populations; however, these benefits have been limited by the impact of other chronic conditions among which Hepatitis C occupies a significant place. Chronic liver disease due to Hepatitis C affects a large number of HIV-infected patients in Latin America and currently poses important challenges for the health systems in the region. Treatment for Hepatitis C infection in monoinfected and dually infected patients has improved dramatically in the past decade. New direct acting antiviral agents are substantially increasing the rate of sustained virological responses observed previously with Peg interferon plus ribavirin regimens specially in difficult to treat patients. Regimens free of Peg interferon more likely will replace the current standard of more toxic and less efficacious options, albeit at costs (if no major changes occur) that are unreachable to most governments. Even though anti-HCV treatment has a limited duration and its goal is viral eradication – a concept not yet existent for HIV – access to anti-Hepatitis C agents has not followed the same path as antiretroviral treatment in the region. In fact, the majority of countries in Latin America do not have specific programs for Hepatitis C treatment access as the ones created to treat HIV infection more than a decade ago. The current era of Hepatitis C treatment in some ways resembles the time in which HAART first became available. Communities, academia, governments and business from the region should learn from the experiences derived from that era in order to face the challenges that Hepatitis C treatment represent. Delaying or negating access now based on purely monetary reasons is not a wise option. Creative solutions to improve access should be sought and implemented in a joint fashion among the different actors.

# Sub article: O341: Resistance measurement: what's on the horizon for clinicians?

**DOI:** 10.7448/IAS.17.2.19208

**Published:** 2014-05-07

**Authors:** Diaz Ricardo Sobhie

**Affiliations:** Infectious Diseases Division, Paulista School of Medicine, Federal University of São Paulo, São Paulo, Brazil

## Abstract

Transmitted drug resistance (TDR) has become an epidemiological and practical issue in any region where that there is widespread use of antiretrovirals. The World Health Organization (WHO) defines low TDR prevalence at a level below 5% and high-prevalence levels above 15%. There is a universal trend to maintain prevalence of NRTI resistance over time, increase NNRTI resistance and decrease PI resistance as a function of viral fitness and increasing use of boosted PIs. Emerging data confirm the relationship between TDR and virologic failure, thus emphasizing the importance of pre-treatment resistance determination. More sensitive methods to determine acquired drug resistance (ADR) using next-generation sequencing are promising and are helping to understand mechanisms of resistance, and also offering a window of opportunity to increase salvage therapy efficacy. Resistance mutants may be selected among low-level viraemic patients on antiretroviral treatment, especially for low genetic barrier drugs. Finally, in the test and treat era, treatment for longer periods of time may lead to long-term toxicity and to treatment interruptions. In this sense, the future points to personalized medicine where genetic tests targeting hosts may help to mitigate chronic side effects of antiretrovirals at an individual level.

# Sub article: O342: Antiretrovirals: new drugs and new strategies

**DOI:** 10.7448/IAS.17.2.19212

**Published:** 2014-05-07

**Authors:** Pozniak Anton

**Affiliations:** HIV Services, Chelsea and Westminster Hospital, London, UK

## Abstract

The search for drugs that are efficacious but have very few side effects, do not induce resistance and are simple in their administration is still a priority in HIV research. The integrase inhibitors have excellent tolerability and low rates of serious adverse events. Recently, the newest integrase inhibitor dolutegravir showed superiority over efavirenz and darunavir/r in randomized naïve studies mainly based on its excellent side effect profile. New drugs in the pipeline include an alternative to tenofovir, as tenofovir alafenamide fumarate (TAF), which may have less bone and renal toxicity. The pharmacokinetic booster cobicistat is used at the moment in Stribild to boost elvitegravir. It is also being co-formulated with darunavir and atazanavir as a single pill to simplify PI therapy. There are prospects of having a single pill “boosted PI regimen.” New drugs in later stages of development with recent data include the attachment inhibitor BMS-663068 from BMS and the long-acting integrase S/GSK744 from ViiV. The experimental entry inhibitor cenicriviroc, a. gne therapy is also being piloted. Nucleoside sparing strategies are still being pursued and a large trial of darunavir/r, and raltegravir in naïve patients (NEAT 001) was non-inferior to standard of care except in those patients with low CD4s and high viral loads. Another smaller study in naïve patients has shown that at least in the short-term, lopinavir plus 3TC was non-inferior to lopinavir plus 2 nucleosides. The use of Maroviroc with DRV/rI has not proven to be efficacious and the MODERN study had to be terminated early.

# Sub article: O343: How are generics shaping ARV use in Latin America?

**DOI:** 10.7448/IAS.17.2.19209

**Published:** 2014-05-07

**Authors:** Ghidinelli Massimo

**Affiliations:** PAHO WHO Regional Office, Washington, DC, USA

## Abstract

This presentation will share the perspective of the Pan American Health Organization (PAHO) on the impact generic antiretrovirals (ARVs) have made in improving access to affordable, quality, safe and efficacious treatment to HIV in Latin America. Increased use of generic medicines, in particular ARVs has demonstrated to be a cost-effective approach and can result in significant reductions in expenditures on ARVs, which has allowed PAHO Member States to accelerate efforts to scale-up treatment towards sustained universal access to ARVs. Examples of how procurement of generic ARVs through the Regional Revolving Fund for Strategic Public Health Supplies (Strategic Fund) has resulted in significant cost savings in Latin America will be discussed. Additionally, the presentation will explore current barriers to accessing generic ARVs, focusing on current patents and licensing agreements between ARV manufacturers that limit the sale of generic ARVs Latin America.

# Sub article: O351: Acute HIV infection

**DOI:** 10.7448/IAS.17.2.19213

**Published:** 2014-05-07

**Authors:** Gulick Roy

**Affiliations:** Weill Medical College of Cornell University, New York, NY, USA

## Abstract

Acute HIV infection is defined as the period of time when the HIV RNA and/or p24 antigen is detectable, but before HIV antibodies are detectable. Early HIV infection is the period of time within six months after infection when HIV antibodies are detectable. The vast majority of HIV infection occurs with R5 virus. The virus crosses a mucosal barrier over 2–6 hours, infects target cells (CD4 lymphocytes, dendritic cells, tissue macrophages) locally and then spreads to local lymph nodes over 3–6 days. After 6–25 days, the infection spreads systemically and peak viraemia occurs; this is associated with a substantial loss of mucosal CD4 cells and the greatest risk of onward transmission. The clinical presentation of acute HIV infections is symptomatic in ~75–90% of patients with non-specific symptoms of fever, myalgias and night sweats; rashes may be present in ~50% and oral ulcers in up to 10–20% of patients. The diagnosis of acute HIV is made with a combination of tests including HIV RNA, p24 antigen (included as part of the 4th generation ELISA test), and HIV antibody testing. Clinical trials of acute HIV infection suggest early antiretroviral treatment is associated with decreased clinical symptoms and signs, enhanced CD4 recovery and possibly a decrease in viral set point and/or HIV reservoirs. Recent studies suggest very early treatment of infection can lead to eventual virologic control of antiretrovirals or possibly even cure in some cases. Current guidelines support treatment, though differ in the strength of the recommendation. With transmitted drug resistance in mind, the optimal treatment choice is a protease inhibitor- (or integrase inhibitor-) based regimen that is started right away and not stopped. Drug resistance testing should be obtained and used to adjust the regimen later, when the results return. Diagnosing and treating acute HIV infection is an effective way to reduce onward HIV transmission.

# Sub article: O352: Progress in HIV cure research

**DOI:** 10.7448/IAS.17.2.19202

**Published:** 2014-05-07

**Authors:** Kuritzkes Daniel

**Affiliations:** Brigham and Women's Hospital, Boston, MA, USA

## Abstract

Research towards a HIV cure has emerged as a major goal of investigators throughout the world, with significant resources being mobilized by national funding agencies and foundations. This renewed sense of purpose is driven in part by the demonstration that cure (or “sustained HIV remission”) may be possible, as illustrated by the Berlin patient and the Mississippi baby. A number of important steps forward in cure research have been reported. The report of a second baby positive at birth but in whom HIV cannot now be detected generated considerable interest at CROI 2014. Because this baby remains on antiretroviral therapy (ART), it is too soon to say whether ART-free remission has been achieved. Preliminary data from an on-going study of the histone deacetylase (HDAC) inhibitor panobinostat show that multiple cycles of the drug resulted in repeated stimulation of HIV expression, extending the results originally reported with vorinostat. In addition, studies in the macaque model of SHIV infection showed that broadly neutralizing monoclonal antibodies reduce the level of viraemia and decrease proviral DNA, suggesting that such antibodies may have a role in combination strategies for reducing the viral reservoir. Treatment interruption in two HIV-infected adults who had received allogeneic stem cell transplants for haematological malignancies and in whom no evidence of viral persistence could be detected in peripheral blood or rectal tissue resulted in virologic rebound after three and eight months. Although disappointing, these results nevertheless suggest an important role for graft-versus-host reaction in eliminating latently infected cells. Important questions that remain to be answered include identifying the site of viral persistence in these patients and determining whether additional interventions can prolong the time to viral relapse.

# Sub article: P1: Acceptability and willingness to receive short message service regarding HIV healthcare information in a HIV adult population in Mexico City

**DOI:** 10.7448/IAS.17.2.19116

**Published:** 2014-05-07

**Authors:** Crabtree-Ramírez Brenda, Caro-Vega Yanink, Sandra G Sosa-Rubi, Kwan Ada, Sierra-Madero Juan, Ortega-Pérez Raul, López-Martínez Alondra, Bautista-Arredondo Sergio

**Affiliations:** 1 Infectious Diseases, Instituto Nacional de Ciencias Médicas y Nutrición, Salvador Zubirán, Mexico City, Mexico; 2 Health Economy, Instituto Nacional de Salud Pública, Cuernavaca, Mexico

## Abstract

**Introduction**: Despite the growing amount of evidence of the effectiveness of short message service (SMS) as a reminder for ARV adherence and health HIV-related educational information, few data have documented the acceptability and willingness to receive this type of services in Latin-American countries. This study aimed to explore the willingness and acceptability to receive SMS among HIV population attending to the Instituto Nacional de Ciencias Médicas y Nutrición, Salvador Zubirán (INCMNSZ) in Mexico City.

**Methods**: We randomly selected 267 patients out of 1500 active patients at the AIDS clinic to apply a questionnaire of 11 items exploring feasibility, acceptability and willingness to receive SMS in different modes and with different contents. Specifically, we explored frequency and content of information regarding HIV healthcare (ARV reminder, clinical visit reminder, information about a healthy life style and emotional support). The study was approved by the Ethics Committee (REF. 899).

**Results**: A total of 267 adult patients were randomly recruited and accepted to participate. Of them, 87% were male, with a median age of 41 years (IQR: 32–47), a median time since HIV diagnosis of 7 years (IQR: 3.2–13.3) and a median time in ARV therapy of four years (IQR: 2.6–9.4). As high as 96% own and use a mobile phone and 68% declared to have received/sent a SMS in the previous day. Only eight patients refused to receive information through SMS. There were no statistical differences between those who accepted and those who refused in terms of gender, HIV viral load undetectability (<40 copies) status and time in ARVs (*p*=0.21, 0.69, 0.44, respectively). Most patients accepted to receive SMS regarding HIV healthcare information once a month (96%) and/or once a week (89%). The content of information willing to be received were “news on HIV research”, followed by “other HIV related diseases”, “nutritional guidance”, “emotional support” and “safe sex information”. No differences were found between willingness to receive such content of information and age, gender, time on ARVs and HIV viral load undetectability status.

**Conclusions**: Acceptability and willingness to receive SMS about HIV healthcare in our population was high regardless of clinical and demographic characteristics. Our results are encouraging and warrant further research on implementation to design an intervention.

## References

[1] da Costa TM, Barbosa BJP, Gomes e Costa DA, Sigulem D, de Fátima Marin H, Filho AC (2012). Results of a randomized controlled trial to assess the effects of a mobile SMS-based intervention on treatment adherence in HIV/AIDS-infected Brazilian women and impressions and satisfaction with respect to incoming messages. Int J Med Inform.

[2] Pop-Eleches C, Thirumurthy H, Habyarimana JP, Zivin JG, Goldstein MP, de Walque D (2011). Mobile phone technologies improve adherence to antiretroviral treatment in a resource-limited setting: a randomized controlled trial of text message reminders. AIDS.

[3] Finitsis DJ, Pellowski JA, Johnson BT (2014). Text message intervention designs to promote adherence to antiretroviral therapy (ART): a meta-analysis of randomized controlled trials. PLoS One.

# Sub article: P2: Public pharmacy data can be used to predict virologic failure for patients on antiretroviral therapy in Brazil

**DOI:** 10.7448/IAS.17.2.19115

**Published:** 2014-05-07

**Authors:** Martin David, Paula M Luz, Magdalena E Sobieszczyk, Jordan E Lake, Jesse L Clark, Dayse P Campos, Valdilea G Veloso, Ronaldo I Moreira, Sandra W Cardoso, Jeffrey D Klausner, Grinsztejn Beatriz

**Affiliations:** 1 College of Physicians and Surgeons, Columbia University, New York, NY, USA; 2 Fundação Oswaldo Cruz, Instituto de Pesquisa Clínica Evandro Chagas, Rio de Janeiro, Brazil; 3 Division of Infectious Disease, Columbia University Medical Center, New York, NY, USA; 4 Department of Medicine, University of California, Los Angeles, CA, USA

## Abstract

**Introduction**: Pharmacy adherence measures such as medication possession ratios (MPRs) have previously been shown to be predictive of virologic outcomes. We aimed to determine the optimal interval of MPR assessment for predicting virologic failure for HIV-infected patients on antiretroviral therapy (ART).

**Materials and methods**: Using national Brazilian ART pharmacy data, we examined MPRs for patients ≥18 years of age with at least one HIV-1 RNA level ≥180 days after ART initiation on or after 1 January 2011. Patients with a documented ART change≤270 days prior to viral load test date were excluded. Logistic regression models were used to describe associations between virologic failure, defined as an HIV-1 RNA level ≥1000 copies/ml and MPRs, defined as number of days index drug dispensed [non-nucleoside reverse-transcriptase inhibitor (NNRTI) or protease inhibitor (PI)] per 180-, 90- and 30-day interval preceding viral load testing. A predictive probability was calculated using the corresponding odds ratios for each MPR interval and the optimal cut-point, maximizing sensitivity and specificity, for predicting the likelihood of virologic failure was selected. Bootstrapping was used to quantify the uncertainty of the performance of the MPR intervals.

**Results**: A total of 1025 patients were included (68% were male, median age 41 years, median time on ART 4.5 years, 57% on an NNRTI). Overall, 9.5% of patients experienced virologic failure. Of the 180-, 90- and 30-day periods, the MPR intervals with the greatest median area under the receiver operating characteristic (AUROC) curve were the 0–180, 90–180 and 150–180 day intervals. At the cutoff of 0.07 for the 180-day interval, 72 out of 89 patients (81%) with virologic failure would be correctly identified based on MPR.

**Conclusions**: The MPR performed well as a predictive tool to identify patients in virologic failure with the 0–180 day interval being marginally more predictive than the 90-day period occurring 90–180 days prior to viral load testing. The validation and use of this predictive tool using public pharmacy data could aid in early identification of patients with poor adherence and prevent development of treatment failure and drug resistance in Brazil.

**Abstract P2–Table 1:** Detailing results for the intervals with the greatest AUROC within the 180-, 90- and 30-day periods

MPR period	180-Day period (0–180)	90-Day period (90–180)	30-Day period (150–180)
Cut-point	0.07	0.08	0.16
AUROC	0.86 [0.82, 0.90]	0.84 [0.80, 0.88]	0.75 [0.71, 0.78]
Sensitivity	81% [73%, 89%]	81% [72%, 89%]	88% [81%, 94%]
Specificity	82% [80%, 84%]	81% [78%, 83%]	58% [55%, 61%]

Detailing results for the intervals with the greatest AUROC within the 180-, 90- and 30-day periods

# Sub article: P3: HIV(+) viral load in individuals with dengue virus infection in the Dominican Republic

**DOI:** 10.7448/IAS.17.2.19119

**Published:** 2014-05-07

**Authors:** Paulino Robert

**Affiliations:** Universidad Iberoamericana, Santo Domingo, Dominican Republic

## Abstract

**Introduction**: Dengue virus (DENV) is a (+) RNA virus that belongs to the flaviviridae family and is transmitted by mosquitoes, primarily *Stegomyia aegypti* [1]. DENV prevalence has grown in the last years in tropical countries in Asia, the Pacific and the Americas [2]. The impact of the co-infection of DENV and HIV is not well described, but some data suggested that in DENV infections, a significant reduction of HIV viral load is observed [3].

**Materials and methods**: A cross-sectional study was conducted in patients with HIV-1 infection in follow-up, with signs and symptoms of DENV infection. DENV infection was confirmed by serological tests (IgG and IgM), at baseline, and after admission to the hospital. CD4+ T cell count was assessed as well as HIV viral load before and three days after admission. Management of DENV infection was done according to the WHO guidelines for dengue management.

**Results**: Six patients with chronic HIV infection and without antiretroviral treatment reported signs and symptoms of DENV infection in a period of two years in an outpatient clinic in Santo Domingo. CD4+ T cells count was assessed as well as HIV viral load at the onset of symptoms. After three days of admission, a plasma sample was obtained. A decrease in HIV viral load was observed in all the cases with a median of 21,532 copies/ml prior DENV infections, and a median of 7624 copies/ml of decrease at third day after admission (SD: 12487.7).

**Conclusions**: The decrease of HIV viral load in plasma reveals an interaction between the immune responses activated against DENV infection, which appears to also be involved in HIV replication. A recent study of *in vitro* study of DENV infection in intentionally infected cells with HIV revealed the role of NS5 replicative proteins [4]. Our study is consistent with these findings.

**Abstract P3–Figure 1:**

## References

[1] Chen LH, Wilson ME (2012). Dengue and chikungunya in travelers: recent updates. Curr Opin Infect Dis.

[2] Halstead SB (2007). Dengue. Lancet.

[3] Watt G, Kantipong P, Jongsakul K (2003). Decrease in human immunodeficiency virus type 1 load during acute dengue fever. Clin Infect Dis.

[4] Lopez U, Vasquez C, Vasquez R, Valle-Reyes S, Guzmán-Bracho C, Araiza-Garaygordobil D (2014). Dengue virus serotype 1 non-structural protein NS5 expression interferes with HIV replication in a CD4+ T-cell line. Am J Trop Med Hyg.

# Sub article: P4: Syphilis diagnosis at point of care provides faster confirmatory results and ensures treatment

**DOI:** 10.7448/IAS.17.2.19118

**Published:** 2014-05-07

**Authors:** Casillas Jesus, González Andrea, Ramos Ubaldo

**Affiliations:** 1 Internal Medicine, Clinica Condesa, Mexico City, Mexico; 2 Mexico City HIV/AIDS Program, Clinica Condesa, Mexico City, Mexico; 3 Obstetrics/Gynaecology, Clinica Condesa, Mexico City, Mexico

## Abstract

**Introduction**: Syphilis diagnosis and treatment at point of care (POC) is essential. CDC's 2013 estimates an annual incidence of 20 million of cases, which account for $16 billion in total costs. Optimizing diagnosis could engage and treat an increased number of infected individuals. Our STI screening includes HIV, VHC, VHB and syphilis tests with reporting time in 10 working days. In CDC's syphilis algorithm, a positive treponema test is followed by VDRL to rule out active syphilis. From January to October 2013, failure to receive STI results in 1516 HIV-negative clients was 74.4% and failure in syphilis results in 896 syphilis cases was 30.7%. The aim of this study was (1) to evaluate the feasibility of conducting syphilis diagnosis at POC comparing three protocols: a) STANDARD: standard treponemal; if positive VDRL, results in 10 working days; b) Treponemal Rapid Test (TPRT): TPRT results in 15 minutes; if positive VDRL results in 10 working days; c) TPRT/VDRL (TPRT/VDRL): TPRT results in 15 minutes; if positive VDRL results in one hour; (2) to evaluate the number and proportion of clients who return to receive results and therapy in every protocol; (3) to monitor the return activity for VHC/VHB tests results.

**Materials and methods**: Clients agreed to take blood samples by signing informed consent, establishing a one week period to compare every protocol. All tests were drawn and processed in the clinic's lab. Study site data and follow-up visit established time to receive results between the blood sampling and the processing and reporting of results. Last date available for returning for results was as 17 January 2014.

**Results:** From 25 November to 13 December 2013, a total of 980 were tested for HIV/STDs at Clinica Condesa (351 STANDARD; 307 TPRT and 322 TPRT/VDRL). Reporting time was 90 minutes in TPRT/VDRL and 10 days in TPRT and STANDARD. Per protocol, the number of received results was superior in TPRT/VDRL (100%) vs. STANDARD (62.8%) and TPRT/VDRL vs. TPRT (64.0%) for syphilis and TPRT/VDRL (43.1%) vs. STANDARD (32.1%) and TPRT/VDRL vs. TPRT (29.3%) for STDs. All results were significant (*p*<0.005). All patients diagnosed with syphilis received therapy.

**Conclusions**: TPRT/VDRL it is the best SOC available: provides fast syphilis diagnosis and ensures opportune therapy, proving to be effective in the rate of returning for syphilis and STI results (36% more) as compared to TPRT alone and STANDARD protocols.

# Sub article: P5: Health survey with seroprevalence in transgender women in Mexico City: factors related to suicidality

**DOI:** 10.7448/IAS.17.2.19123

**Published:** 2014-05-07

**Authors:** Vega Hamid, Colchero Arantxa, Rodríguez Víctor, Hernández Gabriela, Cruz Jeremy, Cortes Alejandra, López Nitzia, Ramírez Claudia, Diaz Steven, González Andrea

**Affiliations:** 1 Mental Health Program, Condesa Specialized Clinic, Mexico City, Mexico; 2 National Institute of Public Health, Cuernavaca, Mexico; 3 Condesa Specialized Clinic, Transgender Clinic, Mexico City, Mexico; 4 Mexico City's HIV/AIDS Program, Condesa Specialized Clinic, Mexico City, Mexico

## Abstract

**Introduction**: Transgender women (TW) have been poorly studied in Mexico. Some qualitative studies suggest that they live in conditions of vulnerability and marginalization throughout their lifetime. These conditions usually result in greater risk of having seriously suicidal thoughts, make a suicidal plan or attempts. One of the main objectives of this study was to assess vulnerable conditions and relate them with TW with history of suicidality in a population-based sample in Mexico City.

**Methods**: This study was part of the first *Health Survey with seroprevalence in Transgender Women in Mexico City*. The survey was implemented in three sets: TW in prison, TW in meeting places and TW at the free care clinic in Mexico City (*Clinica Especializada Condesa*). After signing informed consent, they completed a questionnaire with socio-demographic questions, substance misuse, mental health status, complications from sex generic transformations and barriers related to healthcare access. Suicidality history was measured with WHO's international interview, CIDI 2. Stata/SE 12.0 was used for descriptive analysis, and *t*-test and χ^2^ for comparative analysis between the group with history of suicidality and the negative one.

**Results**: The total sample was 585 TW. Mean age was 32.3±10.1 years old. Only 33% had completed high school or higher education, and 48% were sex workers. HIV prevalence was 33%. Throughout their lifetime, almost 35% had seriously suicidal thoughts, 15% a suicidal plan and 18% had at least one suicidal attempt. Mean of suicidal attempts was three (range=1–35), and mean age of the first one was 19.7±7.7 years old. Factors associated (*p≤*0.01) with suicidality in this sample of TW were having lived on the street ever, have no family support for being TW, have suffered discrimination and have misuse of alcohol or any illegal drugs. Living with HIV was not related to suicidal behaviour (*p=*0.32).

**Conclusions**: TW have, by far, a higher prevalence of suicidality than general Mexican population (2.8%). These findings suggest that poor social support, vulnerable conditions and misuse of substances that TW live throughout their lifetime are associated with suicidality. Mental healthcare systems should be adapted and available to this population to avoid suicidality and improve their quality of life.

## References

[1] Dhejne C, Lichtenstein P, Boman M, Johansson ALV, Langstrom N, Landén M (2011). Long-term follow-up of transsexual persons undergoing sex reassignment surgery: cohort study in Sweden. PLoS ONE.

[2] Borges G, Wilcox HC, Medina-Mora ME, Zambrano J, Blanco J, Walters E (2005). Suicidal behavior in the Mexican National Comorbidity Survey (M-NCS): lifetime and 12-month prevalence, psychiatric factors and service utilization. Salud Mental.

# Sub article: P6: HIV serostatus awareness and associated factors comparing known and unknown HIV sero-positives and HIV sero-negatives among MSM/TW in Lima, Peru

**DOI:** 10.7448/IAS.17.2.19129

**Published:** 2014-05-07

**Authors:** Leon Segundo, Konda Kelika, Jeffrey D Klausner, Coates Thomas, Caceres Carlos

**Affiliations:** 1 UPCH, Public Health, Lima, Peru; 2 UCLA, World Health, Los Angeles, CA, USA

## Abstract

**Introduction**: Lack of HIV status awareness is common among high-risk populations in Peru and can lead to continued risk behaviours. We explored high-risk behaviours among participants of a study that included both HIV testing and reporting of previously known HIV infection.

**Materials and methods**: Data are from a study implemented in Lima, Peru, from 2008 to 2009 assessing HIV prevalence among MSM/TW. The survey collected socio-demographic and risk behaviour data. HIV screening used enzyme immunoassay (EIA) with WB confirmation. HSV-2 testing used an EIA and syphilis testing used an RPR with TPPA confirmation. We conducted separate analyses comparing MSM/TW with known infection to HIV negatives, those with unknown infection to HIV negatives and known to unknown HIV infection. Statistical analyses used Chi-squared, *t*-tests and ANOVA.

**Results**: Of the 714 participants, 131 (18.3%) were HIV positive; among these, 38 (29.0%) knew they were HIV positive, while the remaining 93 (71.0%) were unaware of their HIV infection. People with known HIV infection were significantly older, more likely to have unmet basic needs, more likely to report having only protected insertive anal sex, less likely to report unprotected receptive anal sex and less likely to report using alcohol prior to sex when compared to HIV negatives (all *p*-values <0.01). The only significant differences between participants with unknown HIV infection versus those who were HIV negative was an increase in their number of sexually active years (mean 16.7 vs. 14.9, *p*-value 0.05), HSV-2 (36.7% vs. 22.6%, *p*<0.01), history of syphilis (80.0% vs. 67.8%, *p*=0.04). The comparison of people with known HIV infection to those with unknown HIV infection followed the same patterns as the comparison between people with known HIV infection to those who were HIV negative.

**Conclusions**: Most HIV-infected MSM/TW in Lima, Peru, are unaware of their infection. People unaware of their HIV infection are more involved in risky behaviours that can lead to subsequent HIV transmission to their sex partners as compared to MSM/TW who are aware of their HIV infection. Few factors distinguished participants unaware of their HIV infection status from those who were HIV negative, although ulcerative STIs were more common among MSM/TW with unknown HIV infection. Efforts to promote frequent, periodic HIV testing among high-risk MSM/TW should be improved.

# Sub article: P7: Epidemiological aspects of HIV infection among illicit drug users in the State of Piauí, Northeast Brazil

**DOI:** 10.7448/IAS.17.2.19121

**Published:** 2014-05-07

**Authors:** Oliveira Evaldo, Silva Letiano, Lima Verde Roseane, Aragão Ana Luisa, Castro Jairo, Sawada Leila, Pinto Laine, José Alexandre R Lemos, Aldemir B Oliveira-Filho, Antonio C Vallinoto

**Affiliations:** 1 Center for Health Sciences, Federal University of Piauí, Teresina, Brazil; 2 Health Department of the State of Piauí, Teresina, Brazil; 3 Center for Hematology and Hematology of Pará, Belém, Brazil; 4 Chiba Institute of Technology, Chiba, Japan; 5 Institute of Biological Sciences, Federal University of Pará, Belém, Brazil; 6 Institute for Coastal Studies, Federal University of Pará, Bragança, Brazil

## Abstract

**Introduction**: Illicit drug use is an important public health issue around the world. Much of the estimated burden of disease attributable to the use of illicit drugs is probably due to blood-borne viral infections transmitted by sharing drug use equipment and unprotected sex. Hepatitis B, human immunodeficiency virus and hepatitis C are major health issues among illicit drug users (DUs) [1]. Epidemiological aspects of viral infections among illicit drug users in Northeast Brazil are poorly known. This study determined the prevalence of HIV infection, co-infections HIV-HBV and HIV-HCV and the factors associated with viral infections among DUs in the state of Piauí, Northeast Brazil.

**Materials and methods**: This cross-sectional study of a non-probabilistic convenience sample was based on information and biological samples provided by DUs attended at Central Laboratory and STD/AIDS Reference Unit, both within the Health Department of the State of Piauí. In all samples, the presence of HIV-1/2 was determined by enzyme immunoassay (EIA) and confirmed by Western blot and NASBA. Additionally, HBV, HCV and HDV infection has also been verified using EIA and real-time PCR [2,3]. Simple and multiple logistic regressions were calculated to assess the independent effects of variables. The fit of the final model was assessed using the Hosmer–Lemeshow goodness-of-fit test. All statistical analyses were performed using SPSS 18.0.

**Results**: In total, 243 DUs participated in this study. The majority of participants were male (72.4%). The mean age was 35 years (18–51 years). Most participants (81.1%) reported having consumed more than one illicit drug during their lifetimes. Drug preference was grouped into four categories: cocaine paste (18.1%), cannabis + cocaine paste (32.5%), cocaine powder (13.6%) and oxi cocaine (35.8%). Since, 16 DUs experienced at least once in a lifetime injecting drug. The prevalence of HIV infection among DUs was 44.4%. Being that the prevalence of HBV-HIV and HCV-HIV was 1.6% and 4.9%, respectively. A multivariate analysis identified four risk factors for HIV infection: injecting drug use, unprotected sex, sexual intercourse with another DUs and more than 10 sexual partners in the last year.

**
Conclusions**: This study showed a high prevalence of HIV infection among DUs possibly associated with parenteral and sexual transmission of viruses.

## References

[1] Degenhardt L, Hall W (2012). Extent of illicit drug use and dependence, and their contribution to the global burden of disease. Lancet.

[2] Oliveira-Filho AB, Oliveira EH, Castro JA, Silva LV, Vallinoto AC, Lemos JA (2012). Epidemiological aspects of HCV infection in HIV-infected individuals in Piauí State, Northeast Brazil. Arch Virol.

[3] Oliveira EH, Lima Verde RM, Pinheiro LM, Benchimol MG, Aragão AL, Lemos JA (2013). HBV infection in HIV-infected subjects in the state of Piauí, Northeast Brazil. Arch Virol.

# Sub article: P8: HIV and the skin: frequent dermatosis as cutaneous markers in HIV detection

**DOI:** 10.7448/IAS.17.2.19128

**Published:** 2014-05-07

**Authors:** Cruz Palacios Carlos, Ramos Alamillo Ubaldo, González Andrea

**Affiliations:** 1 Dermatology/STI/HIV Transmission, Secretaría de Salud del Distrito Federal de la ciudad de México/Clínica Especializada Condesa, Mexico City, Mexico; 2 Programme Director of HIV/AIDS in Mexico City Fe, Secretaría de Salud del Distrito Federal de la ciudad de México/Clínica Especializada Condesa, Mexico City, Mexico

## Abstract

**Objective**: 1) Determine the most frequent type of dermatosis in people with HIV/AIDS, 2) Propose as cutaneous markers the most frequent dermatosis observed during regular medical visits and thus recognize HIV infections for their opportune treatment and 3) Determine the most common sexually transmitted infections (STIs) in this population group.

**Methodology**: Prospective observational and descriptive study that took place at Condesa Specialized Clinic in Mexico City between August 2005 and November 2013, in 1200 patients with HIV infection and evaluated during their first visit by a dermatologist, emphasizing on the diverse dermatosis and STIs during the consultation. The following variables from the clinical file were analyzed: age, gender, education, sexual activity initiation (SAI), condom use, number of partners; clinical/lab diagnosis (Serology: VDRL, anti-treponema antibodies, Hepatitis B and C; gonorrhoea, PCR for *Chlamydia trachomatis*, KOH and skin biopsy) and the relationship with CD4 count. All services were offered in a respectful and confidential manner, respecting their human rights.

**Results**: A total of 1200 patients with HIV/AIDS were studied, all men who have sex with men (HSH); with HIV 422 (35.17%) (CDC: A1, B1 y C1 B2) and with AIDS 778 (64.83%) (CDC: A3, B3, C3). Average age 29 years (range 16–74 years). Education: primary 14.9% and university (9.6%) (Degree), Average SAI: 16 years, avg. number of sexual partners: 34 in three years, avg. age of sexual abuse: seven years. Condom use 57.7% and for oral sex 7.4%, with an average of years from an HIV diagnosis. The initial dermatosis was registered on average two years after receiving an HIV diagnosis and one year if the diagnosis was AIDS. The five more frequent dermatosis in patients with HIV out of 15 observed were: seborrheic dermatitis 30.9% (371), HIV-related pruritic papular dermatitis (PPD) 29.25% (351), xerosis disseminated 29% (348), painful pruritic folliculitis 22.7% (272) and oral candidiasis 20.1% (242). In patients with AIDS: oral candidiasis 31.42% (377), atopic dermatitis disseminated 28.2% (338), onychomycosis 22.6% (271), seborrheic dermatitis 18% (216) y PPD 17.8% (213). The five more common STIs out of eight researched in HIV patients were perianal condylomata acuminata (PAAC) 26.8% (322), molluscum contagiosum (MC) 22% (269), genital herpes simplex 16.1% (193), warts in coronal sulcus 10.8% (130) and late latent syphillis (LLS) over two years 8.17% (98). With AIDS patients: MC 20.5% (246), PAAC 16.75% (201), genital herpes simplex 12.9% (155), warts in coronal sulcus 11.17% (134) and LLS 2.75% (33). Average CD4 count: patients with HIV: 349.5 and patients with AIDS less than 200.

**Conclusions**: Dermatosis are frequent among people with HIV/AIDS, with the scaly and itchy ones the most common and the tumour-related STIs, such as CAAP, syphillis and molluscum contagiosum, which would suggest that for a patient presenting this dermatopathy an HIV test is strongly recommended.

# Sub article: P9: Epidemiological aspects of HIV-1 infection amongst illicit drug users in the Marajó Archipelago, Brazilian Amazon

**DOI:** 10.7448/IAS.17.2.19130

**Published:** 2014-05-07

**Authors:** Aldemir B Oliveira-Filho, Pacheco Suzy, Pinheiro Luiz Marcelo, Hermes Renata, Amaral Carlos Eduardo, Luciana MC Maradei-Pereira, Crescente Jose Angelo, José Alexandre R Lemos

**Affiliations:** 1 Institute for Coastal Studies, Federal University of Pará, Bragança PA, Brazil; 2 Faculty of Natural Sciences, Federal University of Pará, Campus Marajó, Breves PA, Brazil; 3 Laboratory of Molecular Biology, Center for Hematology and Hematology of Pará, Belém, PA, Brazil; 4 Nucleus of Tropical Medicine, Federal University of Pará, Belém, PA, Brazil; 5 Institute of Biological Sciences, Federal University of Pará, Belém, PA, Brazil

## Abstract

**Introduction**: Latin America contains the third highest estimated number of people living with HIV-1 in the world [1]. Illicit drug users (DUs) are vulnerable to HIV and other blood-borne pathogens as a result of sharing contaminated syringes and other equipment [1]. Epidemiological aspects of HIV-1 infection in the Brazilian Amazon are poorly known, especially in risk groups. This study determined the prevalence and factors associated with infection by HIV-1 among DUs in the Marajó Archipelago, Brazilian Amazon.

**Materials and methods**: This cross-sectional study of a non-probabilistic convenience sample was based on information and biological samples provided by DUs in an area of intense illicit drug use located in the municipalities of Anajás, Bagre, Breves, Curralinho, Gurupá, Melgaço, Ponta de Pedras, São Sebastião da Boa Vista, Salvaterra and Soure, all from the Marajó Archipelago, Brazilian Amazon. DUs were sampled using the snowball technique (July 2012 to December 2013) [2]. In all samples, the presence of HIV-1 was determined by enzyme immunoassay (EIA) and confirmed by Western blot. Additionally, HCV infection has also been verified using ELISA and real-time PCR. Simple and multiple logistic regressions were calculated to assess the independent effects of variables. The fit of the final model was assessed using the Hosmer–Lemeshow goodness-of-fit test. All statistical analyses were performed using SPSS 18.0.

**Results**: In 466 DUs, most participants (73.2%) reported having consumed more than one illicit drug during their lifetimes. Drug preference was grouped into five categories: cannabis (23.8%), cocaine paste (7.9%), cannabis+cocaine paste (25.1%), cocaine powder (15.9%) and oxi cocaine (27.3%). Since, 92 DUs experienced at least once in a lifetime injecting drug. Table 1 shows the demographic and epidemiological characteristics of study participants. In 466 DUs, 165 (35.4%) were identified with HIV-1. The prevalence of co-infections with HIV–HCV was 9.4%. A multivariate analysis identified seven risk factors for HIV-1 infection (Table 1).

**Abstract P9–Table 1:** Demographic and epidemiological characteristics of illicit drug users in the Marajó Archipelago, Brazilian Amazon

Characteristics	Overall sample, N (%)	HIV+ n (%)	OR (CI 95%)
Total	466	165	–
Male	317 (68.0)	109 (66.1)	0.9 (0.6–1.3)
Heterosexual	420 (90.1)	151 (91.5)	1.0 (0.5–2.1)
Age>35 years	140 (30.0)	104 (63.0)	12.6 (7.8–20.1)
HCV+	132 (28.3)	44 (26.7)	0.9 (0.6–1.3)
Supposed route of HIV-1 infection			
Recipient of blood transfusion	73 (15.7)	30 (18.2)	1.3 (0.8–2.2)
Surgery	152 (32.6)	50 (30.3)	0.8 (0.6–1.3)
Tattoos	289 (62.0)	104 (63.0)	1.1 (0.7–1.6)
Injecting drug use	92 (19.7)	82 (49.7)	28.7 (14.3–56.9)
Shares of paraphernalia during drug user	286 (61.4)	111 (67.3)	1.5 (0.9–2.2)
Uses drugs for more than three years	270 (57.9)	132 (80.0)	4.7 (3.0–7.3)
Incarceration	114 (24.5)	38 (23.0)	0.9 (0.6–1.4)
Unprotected sex	391 (83.9)	156 (94.5)	4.9 (2.4–10.0)
Sexual intercourse with other illicit drug user	317 (68.0)	139 (84.2)	3.7 (2.3–5.9)
Involvement in prostitution	236 (50.6)	96 (58.2)	1.7 (1.1–2.3)
More than 10 sexual partners in last 12 months	228 (48.9)	141 (85.4)	14.5 (8.7–23.8)
Risk factors of HIV-1 infection (results of multiple logistic regression)*			
Age>35 years	140 (30.0)	104 (63.0)	11.8 (7.5–20.1)
Injecting drug use	92 (19.7)	82 (49.7)	17.1 (11.2–27.4)
Uses drugs for more than three years	270 (57.9)	132 (80.0)	4.5 (2.0–5.8)
Unprotected sex	391 (83.9)	156 (94.5)	5.6 (1.9–11.3)
Sexual intercourse with other illicit drug user	317 (68.0)	139 (84.2)	4.1 (2.4–8.5)
Involvement in prostitution	236 (50.6)	96 (58.2)	2.3 (1.8–6.2)
More than 10 sexual partners in last 12 months	228 (48.9)	141 (85.4)	15.2 (9.4–26.3)

* The Hosmer-Lemeshow test indicated that the model is well adjusted.

Demographic and epidemiological characteristics of illicit drug users in the Marajó Archipelago, Brazilian Amazon

The Hosmer-Lemeshow test indicated that the model is well adjusted.

**Conclusions**: This study provided important information on HIV-1 infection among DUs that can be used for directing strategies and policies for prevention and control of this infection in this risk group and in the general population.

## References

[1] Strathdee SA, Stockman JK (2010). Epidemiology of HIV among injecting and non-injecting drug users: current trends and implications for interventions. Curr HIV/AIDS Rep.

[2] Oliveira-Filho AB, Sawada L, Pinto LC, Locks D, Bahia SL, Brasil-Costa I (2013). HCV infection among cocaine users in the state of Pará, Brazilian Amazon. Arch Virol.

# Sub article: P10: Clinical and epidemiological characteristics of HIV infection in Latin-American immigrants in a clinical centre in Santiago, Chile, in the last decade

**DOI:** 10.7448/IAS.17.2.19120

**Published:** 2014-05-07

**Authors:** Rodriguez Maria Fernanda, Wolff Marcelo, Claudia P Cortes

**Affiliations:** 1 Infectious Diseases Department, Fundacion Arriaran, Santiago, Chile; 2 Internal Medicine, Universidad de Chile, Santiago, Chile

## Abstract

**Introduction**: There has been an increasing number of immigrants to Chile in the last years, especially from South American countries. The phenomenon of immigration and its consequences has been studied by international literature, and different healthcare needs have been reported for this group as compared with local population. In Chile, this phenomenon is poorly studied and HIV prevention campaigns are focused on national population needs. The purpose of these study is to determine clinical and epidemiological characteristics of the HIV infection in Latin-American immigrants presenting to a referral HIV clinical care centre between the years 2003 and 2013 and bring up the need for development of different prevention strategies focusing in this groups special needs.

**Methods**: A retrospective analysis was conducted. The baseline characteristics of Latin-American immigrants at admission to the infectious disease unit were compared to a peered group of Chileans in the same unit. Recollection of data included clinical variables: CDC stage, sexually transmitted diseases, AIDS defining disease(s), current status; socio-demographic variables: country of origin, gender, schooling, date of birth, use of drugs, tobacco and alcohol; variables related to HIV diagnose: date of confirmation, date of admission, mode of infection; laboratory tests: baseline CD4+, serologies for toxoplasmosis, B and C hepatitis, Chagas and syphilis, PPD; others such as HAART adherence.

**Results**: A total of 187 Latin-American immigrants where compared to 174 Chilean patients. There was an increase in the number of immigrants throughout the observation period. There were no differences in clinical presentation. Foreigners presented larger proportion of women (25% vs. 10%, *p*<0.0001) and heterosexual conduct as compared to nationals (36% vs. 22%, *p*<0.0099). The majority of immigrants came from Peru (51%) and Colombia (11%). In both groups, there was a high percentage of patients with reported sexually transmitted diseases (STDs) previous to HIV diagnose (45% for Chileans and 37% for immigrants).

**Conclusions**: Clinical and epidemiological presentation in foreigner and native population was similar except for gender and sexual behaviour. This brings up the need to address different prevention strategies with more emphasis in women and heterosexual population in this vulnerable group.

# Sub article: P11: Sexually transmitted infections among female sex workers in riverside communities in the Marajo Archipelago, Brazilian Amazon

**DOI:** 10.7448/IAS.17.2.19126

**Published:** 2014-05-07

**Authors:** Pinheiro Luiz Marcelo, Denise Jf Lima, Amaral Carlos Eduardo, Luciana MC Maradei-Pereira, José Alexandre R Lemos, Aldemir B Oliveira-Filho

**Affiliations:** 1 Federal University of Pará, Faculty of Natural Sciences, Breves-Pará, Brazil; 2 Center for Hematology and Hematology of Pará, Molecular Biology, Belém-Pará, Brazil; 3 Center for Hematology and Hematology of Pará, Hematology, Belém-Pará, Brazil; 4 Federal University of Pará, Institute of Biological Sciences, Belém-Pará, Brazil; 5 Federal University of Pará, Institute for Coastal Studies, Bragança-Pará, Brazil

## Abstract

**Introduction**: Sexually transmitted infections (STIs) represent a considerable public health problem, and their prevalence is greater among populations that exhibit high-risk behaviours, such as female sex workers (FSWs). Many FSWs use illicit drugs and have sex without condoms [1]. In Brazilian Amazon, epidemiological studies on STIs among FSWs are scarce. This study determined the prevalence of HIV-1, HTLV-1/2, HBV and *Treponema pallidum* among FSWs located in riverside communities in the Marajo Archipelago.

**Materials and methods**: This cross-sectional study of a non-probabilistic convenience sample was based on information and biological samples provided by FSWs in prostitution spots on riverside communities Antonio Lemos, Capinal, Ramex, São Francisco (Tajapurú River), Intel e Magebras (Mearim River), São Benedito (Jacaré Grande River) and Mainard (Jaburu River). FSWs were sampled using the snowball technique (March 2011 to December 2013). The characteristics of FSWs were collected through a structured questionnaire. The presence of HIV-1, HTLV-1/2 and HBV was determined by enzyme immunoassay and confirmed by real-time PCR. *T. pallidum* has also been verified using VDRL and real-time PCR. Associations between STIs and possible risk factors were assessed using Fisher's Exact Test. Furthermore, odds ratio and 95% confidence intervals were constructed.

**Results**: In total, 89 FSWs participated in this study. The average age was 26.5 years (18–49 years). The average working time was 3.5 years (1–21 years). Most reported they have had first intercourse from 11 years old (59.6%). All of them reported they have been sexually abused. In addition, 52.8% reported having fixed sexual partners (no-clients) and 24.8% reported having already done abortion. The majority (67.4%) serves at least 15 clients per week. Being that the price charged for sex varies from R$10 to R$150. In 89 FSWs, 13 had STIs. The prevalence of HIV-1, HTLV-1, HTLV-2, HBV and *T. pallidum* was 2.2% (2/89), 2.2% (2/89), 1.1% (1/89), 2.2% (2/89) and 9.0% (8/89), respectively. Two FSWs were diagnosed with HIV–HBV co-infection. Three risk factors for STIs were identified (Table 1).

**Abstract P11–Table 1:** Epidemiological characterization of female sex workers in riverside communities in the Marajo Archipelago, Brazilian Amazon

Characteristic	FSWs	STIs+ (%)	*p*-value	OR (95% IC)
Age over 30	29	3 (10.3)	0.32	0.6 (0.2–2.3)
More than eight years of schooling	28	4 (14.3)	0.61	0.9 (0.3–3.4)
Uses illicit drugs (inhaled)	26	2 (7.7)	0.19	0.4 (0.1–1.9)
Carries out periodic gynaecological examination (last three years)	15	2 (13.3)	0.62	0.9 (0.2–4.5)
More than three years as a sex worker	32	8 (25.0)	0.04	3.5 (1.1–11.7)
More than R$ 50.00 per sex (last year)	51	11 (21.6)	0.03	4.9 (1.1–23.9)
More than five sexual encounters paid per day (last year)	6	1 (16.7)	0.59	1.2 (0.1–11.0)
More than 15 clients per week (last year)	21	3 (14.3)	0.64	1.0 (0.2–3.9)
Uses condoms during sexual intercourses with partners	46	4 (8.7)	0.17	0.4 (0.1–1.6)
Uses condoms during sexual intercourses with clients	48	4 (8.3)	0.07	1.9 (0.5–7.3)
Dispenses the use of condoms when knowing the client	18	4 (22.2)	0.25	1.9 (0.5–7.3)
Dispenses the use of condoms when in great necessity of cash	45	11 (24.4)	0.01	6.8 (1.4–32.7)
Dispenses the use of condoms when having sex for many days	15	3 (20.0)	0.38	1.6 (0.4–6.7)

Epidemiological characterization of female sex workers in riverside communities in the Marajo Archipelago, Brazilian Amazon

**Conclusions**: This study showed the features of FSWs who work in rivers of the Marajo Archipelago (Brazilian Amazon), and it identified that the necessity for cash facilitates the transmission of microorganisms during intercourse.

## References

[1] Damascena GN, Szwarcwald L, Souza Junior PRB, Dourado I (2011). Risk factors associated with HIV prevalence among female sex workers in 10 Brazilian cities. J Acquir Immune Defic Syndr.

# Sub article: P12: Mexico City's HIV/AIDS program: a community-based approach to control of an epidemic

**DOI:** 10.7448/IAS.17.2.19122

**Published:** 2014-05-07

**Authors:** Diaz Steven, González Andrea, Gras Nathalie

**Affiliations:** 1 HIV/AIDS Program, Condesa Clinic, Mexico City, Mexico; 2 HIV/AIDS Program, Clinica Especializada Condesa, Mexico City, Mexico

## Abstract

**Introduction**: Mexico has a concentrated HIV epidemic among the following groups: men who have sex with men, male sex workers, transgender women, injection drug users and female partners of men with HIV [1]. Mexico City has the oldest and largest epidemic in the country with a prevalence of 0.78% [2] vs. 0.38% [3] for the country. At the end of 2013, 9114 patients were receiving care at the Condesa Specialized Clinic (CEC), the largest single HIV clinic in Latin America.

**Method**: In order to respond to a challenging epidemic concentrated in several risk groups requires a multi-institutional approach. The CEC was a response to the community pressure to act on a growing epidemic in the city. Since its inception, CEC has had a symbiotic relationship with academia and civil society. Prior to HIV treatment universal access in Mexico, civil society and research institutes helped provide treatment to people with HIV. With HIV universal treatment access in the country, the HIV/Aids Program created linkages with key players from other sectors for the creation of programs to address the needs of specific population groups.

**Results**: The collaboration with civil society and academia has produced several effective programs linked to HIV detection, HIV retention, treatment adherence and prevention promotion. Specific programs such as the HIV Program in prison settings have helped reduce the mortality rate of HIV in prisons from 11% to less than 1%. The Women's program provides specific care to women with HIV, including prenatal care. The Program for Male Sex Workers is a collaboration with academia and started as a research project. This group has an HIV prevalence of 35% with high levels of drug use. Treatment desertion was reduced from 2012 to 2013 by more than 30% at CEC thanks to a collaboration with civil society. The change of access to services and the expediency of in-house lab services increased HIV+ detection 45% in 2013 to 3171 new cases. The installation of the “Test and Treat” modality as part of a cascade process of care (Figure 1) achieved a reduction of time of four and a half months to reach undetectable viral load in patients.

**Abstract P12–Figure 1:**

**Conclusions**: The control of the HIV pandemic can only be achieved with a multisectorial approach. Each sector has tools and competencies that can contribute towards the reduction of HIV incidence and improve the quality of life of people with HIV.

## References

[1] Censida (2012). El VIH/Sida en México.

[2] Report from the Research Center for STI (CIITS) (2010).

[3] UNAIDS (2010). Global Report.

# Sub article: P14: Sofosbuvir plus ribavirin for HCV genotype 1-3 infection in HIV co-infected patients (PHOTON-1)

**DOI:** 10.7448/IAS.17.2.19133

**Published:** 2014-05-07

**Authors:** Rodriguez-Torres Maribel, Naggie Susanna, Sulkowski Mark, Lalezari Jacob, Fessel W Jeffrey, Mounzer Karam, Shuhart Margaret, Luetkemeyer Annie, Asmuth David, Gaggar Anuj, Ni Liyun, Svarovskaia Evguenia, Symonds William, McHutchison John, Dieterich Douglas

**Affiliations:** 1 Fundacion De Investigacion San Juan, Infectious Diseases, San Juan, Puerto Rico; 2 Clinical Research, Duke Clinical Research Institute, Durham, NC; 3 Johns Hopkins University, Viral Hepatitis Center, Baltimore, MD; 4 Clinical Research, Quest Clinical Research, San Francisco, CA, USA; 5 HIV Research Unit, Kaiser Permanente, San Francisco, CA, USA; 6 Philadelphia FIGHT, The Jonathan Lax Treatment Center, Philadelphia, PA, USA; 7 Viral Hepatitis and Liver Clinic, Harborview Medical Center, University of Washington, Seattle, Washington, DC, USA; 8 Department of Medicine, San Francisco General Hospital, University of California, San Francisco, CA, USA; 9 Internal Medicine, University of California Davis Medical Center in Sacramento, Sacramento, CA, USA; 10 Clinical Research–Liver Disease, Gilead Sciences, Foster City, CA, USA; 11 Biostatistics, Gilead Sciences, Foster City, CA, USA; 12 Clinical Virology, Gilead Sciences, Foster City, CA, USA; 13 Clinical Research, Gilead Sciences, Foster City, CA, USA; 14 Clinical Exec–LVD, Gilead Sciences, Foster City, CA, USA; 15 Division of Liver Diseases, Mount Sinai School of Medicine, New York, NY, USA

## Abstract

**Introduction**: Interferon-free treatments for HCV that can be safely co-administered with antiretroviral therapy (ART) are needed for HIV/HCV co-infected patients. We evaluated the safety and efficacy of sofosbuvir (SOF), a pan-genotypic HCV NS5B inhibitor, with ribavirin (RBV) in HCV genotype (GT) 1–3 patients co-infected with HIV.

**
Methodology**: HCV patients with stable HIV disease received SOF 400 mg QD and RBV 1000–1200 mg/day; treatment-naïve GT 1 and treatment experienced GT 2/3 patients received 24 weeks and treatment naïve GT 2/3 patients received 12 weeks of treatment. Multiple ART regimens were permitted as were patients with compensated cirrhosis. The primary efficacy endpoint was sustained virologic response 12 weeks after treatment, (SVR12); safety assessments included HIV RNA and CD4 cell levels.

**Results**: Baseline characteristics and virologic responses are shown in the table. Among treatment naïve GT 2 and 3 patients, SVR12 was achieved in 88% (23/26) and 67% (28/42) respectively. Among the 13 total virologic failures in these groups, only one had on-treatment virologic breakthrough due to study drug non-compliance. No S282T resistance mutations have been detected from virologic failures to date. Complete SVR24 results for all groups, including treatment-experienced GT 2 and GT 3 patients, will be presented. In all groups, treatment discontinuations due to adverse events (AEs) have been uncommon (3%) and grade 3/4 AEs were reported in 25 (11%) patients. Two patients had HIV breakthrough: one in the setting of ART non-adherence, and one regained HIV control without ART change.

**Conclusions**: Treatment-naïve HCV GT 2 and 3 patients co-infected with HIV achieved high rates of SVR12 an interferon-free, all-oral regimen of SOF+RBV. These data suggest that SOF+RBV treatment was well-tolerated and safely co-administered with multiple ART regimens and may be equally safe and efficacious in patients with and without HIV co-infection.

**Abstract P14–Table 1:** 

	GT 1 TN	GT 2 TN	GT 3 TN	GT 2 TE	GT 2 TE
	N=114	N=26	N=42	N=24	N=17
Baseline Characteristics					
Male, n (%)	93 (82)	21 (81)	34 (81)	23 (96)	14 (82)
Black, n (%)	37 (33)	6 (23)	2 (5)	6 (25)	1 (6)
IL28B CC genotype, n (%)	30 (27)	10 (39)	15 (36)	10 (42)	10 (59)
Cirrhosis, n (%)	5 (4)	1 (4)	6 (14)	4 (17)	6 (35)
Log10 HCV RNA (IU/ml), mean (SD)	6.6 (0.8)	6.5 (0.6)	6.2 (0.6)	6.5 (0.8)	6.4 (0.5)
CD4 T-cell count (cells/ml), mean (SD)	636 (251)	627 (278)	559 (224)	649 (330)	671 (346)
On ART, n (%)	112 (98)	22 (85)	39 (93)	23 (96)	16 (94)
ART Regimen:					
Tenofovir/Emtricitabine PLUS					
Efavirenz, n (%)	42 (37)	7 (27)	13 (31)	9 (39)	7 (44)
Atazanavir/ritonavir, n (%)	24 (21)	4 (15)	3 (7)	5 (22)	3 (19)
Darunavir/ritonavir, n (%)	15 (13)	6 (23)	11 (26)	0	2 (12)
Raltegravir, n (%)	21 (18)	2 (8)	6 (14)	4 (17)	3 (19)
Other, n (%)	10 (9)	3 (12)	6 (14)	6 (26)	1 (6)
SVR12, n/N (%)	87/114 (76)	23/26 (88)	28/42 (67)	To be presented	To be presented

# Sub article: P15: Large and small artery elasticity in well-controlled HIV patients

**DOI:** 10.7448/IAS.17.2.19137

**Published:** 2014-05-07

**Authors:** Kundro Mariana, Viloria Guillermo, Toibaro Javier, Losso Marcelo

**Affiliations:** HIV Unit, Hospital J.M. Ramos Mejia, Buenos Aires, Argentina

## Abstract

**Introduction**: HIV infection is associated with increased cardiovascular (CV) risk. Arterial elasticity assessment is a marker of early CV disease. We aim to compare arterial elasticity assessments in HIV-infected patients in virological suppression and non-infected subjects.

**Methods**: Large and small artery elasticity (LAE and SAE) were assessed by analysis of radial pulse waveform. A single set of measurements were performed in HIV-infected patients on stable ART with HIV RNA <50 cp/ml for at least one year and seronegative subjects. Patients with prior cardiovascular events, arritmias, pregnancy and systemic vasculitis were excluded. Univariate associations were analyzed using Spearman's correlation coefficients and multivariate linear regression to determine independent correlates of arterial elasticity.

**Results**: A total of 70 HIV-infected patients and 50 seronegative subjects were included. There were no differences between groups regarding to age (median 39 vs. 36 years), male sex (60 vs. 62%), BMI (24 vs. 25 kg/m2), smoking status (43% vs. 50% smokers), arterial hypertension (4% vs. 9%) and dyslipemia (29% vs. 12%) For HIV-infected patients, the median time from diagnosis was eight years with a median time of 60 months on ART (IQR 27–108 months). As high as 76% of them were on NNRTIs and 24% on PI-based regimens with a median CD4+ count of 539 cell/mm We found no differences neither in LAE (median 16.17 ml/mmHg×10 for HIV patients vs. 14.9 ml/mmHg×10 for seronegative subjects, *p*=0.44) nor in SAE assessments (median 6.73 ml/mmHg×100 vs. 7.13 ml/mmHg×100, *p*=0.59). LAE was inversely associated with older age (*p*=0.05), high levels of total cholesterol (*p*=0.009) and cumulative exposure to ART (*p*=0.004), whereas SAE was only inversely associated with older age (*p*=0.03). Time since HIV diagnosis and CD4+ count bore no association with arterial elasticity in HIV patients.

**Conclusions**: Well-controlled HIV infection was not associated with impaired arterial elasticity in this cohort. Our data is consistent with some previous studies assessing arterial stiffness. In addition to traditional risk factors, the time of exposure to antiretroviral therapy appears to be associated with detriment of arterial elasticity.

## References

[1] Echeverría P, Bonjoch A, Moltó J, Jou A, Puig J, Ornelas A (2014). Pulse wave velocity as index of arterial stiffness in HIV-infected patients compared with a healthy population. J Acquir Immune Defic Syndr.

[2] Lekakis J, Ikonomidis I, Palios J, Tsiodras S, Karatzis E, Poulakou G (2009). Association of highly active antiretroviral therapy with increased arterial stiffness in patients infected with human immunodeficiency virus. Am J Hypertens.

[3] Baker JV, Duprez D, Rapkin J, Hullsiek KH, Quick H, Grimm R (2009). Untreated HIV infection and large and small artery elasticity. J Acquir Immune Defic Syndr.

[4] Sevastianova K, Sutinen J, Westerbacka J, Ristola M, Yki-Järvine H (2005). Arterial stiffness in HIV-infected patients receiving highly active antiretroviral therapy. Antivir Ther.

# Sub article: P16: High-risk HPV genotypes and anal and cervical dysplasia among HIV(+) women, Dominican Republic

**DOI:** 10.7448/IAS.17.2.19136

**Published:** 2014-05-07

**Authors:** Paulino Robert, Garrido Luis, Fernandez Alejandro, Gomez Natalia, Tejada Jean Carlos

**Affiliations:** 1 Research, Universidad Iberoamericana, Santo Domingo, Dominican Republic; 2 School of Medicine, Universidad Iberoamericana, Santo Domingo, Dominican Republic; 3 Research, Instituto Dominicano de Estudios Virologicos, Santo Domingo, Dominican Republic

## Abstract

**Introduction**: Human papilloma virus (HPV) has been linked to anal, penile and cervical cancers [1]. HPV can be classified as highly oncogenic or low-oncogenic depending on strains related to cellular modifications induced by direct invasion of basal membrane in skin. When compared with HIV-uninfected women, women with HIV have higher rates of HPV infection and cervical dysplasia [2, 3].

**Materials and methods**: Participants were selected depending on past clinical history of cervical cytological modifications. All patients were screened with cervical and anal samples with cytobrush and molecular analysis with HPV genotype assays.

**Results**: Fifteen percent reported having anal sex with their sexual partners. Retro-prospective and prospective analysis of cervical Pap smear results revealed cervical intraepithelial neoplasia (CIN) ranging from I to III, CIN-I (n=52), CIN-II (n=32) and CIN-III (n=16). Those findings were detected in a mean of one year. Cytological analysis of anal samples reported six cases of Anal Intraepithelial Neoplasia (AIN) grade I (n=6) and/or koilocytes (n=14); the correlation of AIN-I and Koilocytes was in a ratio of 6:14. Of these 14 cases, only one reported having anal intercourse. In the molecular analysis, we found that HPV16 was detected in 35 cases in either CIN-III/II/I, HPV18 in 21 cases of CIN, HPV45 was detected in six cases of CIN and four in AIN-I/Koilocytes, meanwhile HPV51, 52 and 68 were only detected among those with AIN-I/Koilocytes in anal epithelium.

**Conclusions**: Anal and cervical comparison in HIV-infected patients reveals a correlation of cellular modifications induced by HPV infections. The finding that only one of 20 cases with HPV-related modifications (Koilocytes/AIN) reported having anal intercourse strongly suggests that not only anal intercourse must be considered as a mode of transmission of HPV, skin to skin contact within anal epithelium may confer a suitable mode of transmission in those with cervical or genital HPV infections. This is in addition to anal intercourse as a mode of transmission. Is also noted, a specific preference by some HR-HPV, specifically HPV45, HPV51 and HPV52.

**Abstract P16–Figure 1:** Distribution of HR-HPV Genotypes in Anal Samples.

Distribution of HR-HPV Genotypes in Anal Samples.

## References

[1] Palefsky JM (1998). Human papillomavirus infection and anogenital neoplasia in human immunodeficiency virus-positive men and women. J Natl Cancer Inst Monogr.

[2] Goodman MT, McDuffie K, Hernandez BY, Wilkens LR, Zhu X, Thompson PJ (2011). The influence of multiple human papillomavirus types on the risk of genotype-concordant incident infections of the anus and cervix. J Infect Dis.

[3] Goodman MT, Shvetsov YB, McDuffie K, Wilkens LR, Zhu X, Ning L (2008). Acquisition of anal human papillomavirus (HPV) infection in women: the Hawaii HPV Cohort study. J Infect Dis.

# Sub article: P17: Value of the addition of a treponemal test (FTA-Abs) in the routine serologic screening for syphilis in an HIV-infected cohort

**DOI:** 10.7448/IAS.17.2.19135

**Published:** 2014-05-07

**Authors:** Chatziastros Panagiotis, Chrysou Konstantina, Friligou Eleni, Mistylis Panagiotis, Mpakalis Ioannis, Paraskeva Dimitra, Karageorgopoulos Drosos, Chrysos Georgios

**Affiliations:** 1 Tzaneio General Hospital of Piraeus, Infectious Disease Unit, Athens, Greece; 2 Hellenic Center for Disease Control and Prevention, HIV Office, Athens, Greece

## Abstract

**Introduction**: The diagnosis of syphilis can be complicated in HIV+ patients because of false-negative and false-positive serological tests. A negative Rapid Plasma Reagin (RPR) test result may not rule out syphilis, particularly late latent syphilis. The purpose of this study was to evaluate the utility of both Fluorescent Treponemal Antibody Absorption (FTA-ABS) and RPR for the diagnosis of syphilis in HIV+ patients, driven by an increasing number of syphilis cases during the last two years.

**Methods**: Serum samples from 184 out of the 350 HIV+ patients followed in our outpatient HIV Clinic were tested using both RPR and FTA-ABS to routinely screen for asymptomatic syphilis in sexually active patients on a periodic basis (every 6–12 months).

**Results**: In 145/184 samples (78.80%), results were negative with both methods, excluding syphilis. In 26/184 (14.13%), FTA-ABS was positive, while 12/26 (46.15%) of these were RPR+ and 14/26 (53.85%) RPR−; only five of these 14 patients had a history of treated syphilis. In additional 13/184 patients (7.07%), FTA-ABS was weakly positive, while in nine of them RPR was negative and in four positive (early symptomatic syphilis). There was no case of a false-positive RPR test. Although the sensitivity of RPR in diagnosing syphilis is considered very high, case reports have rarely described RPR-seronegative syphilis in HIV+ patients. A negative treponemal test may not rule out syphilis in patients with advanced immunosuppression. Reinfection may be difficult to rule out in some patients, and reactivation or relapse of a previously treated infection is also possible in an HIV+ person. Positive FTA-ABS with RPR+ resulted in confirmation of syphilis. The diagnostic value of FTA-ABS is greater in HIV+ patients with latent syphilis. FTA-ABS turns positive earlier and remains positive longer than RPR.

**Conclusions**: At screening, positive or weakly positive serological markers of syphilis need a confirmatory test with another treponemal test. Clinicians should consider the sensitivity of FTA-ABS to be high in HIV-infected persons with untreated syphilis beyond the primary stage of infection. If asymptomatic patients have a positive non-treponemal test and negative confirmatory treponemal test results, it is very unlikely that they have active syphilis.

# Sub article: P18: Multiple resistance and unusual mutations from HIV-1 infected Peruvian patients with highly active antiretroviral therapy

**DOI:** 10.7448/IAS.17.2.19117

**Published:** 2014-05-07

**Authors:** Yabar Carlos, Acuña Maribel, Salinas Gabriela, Edgardo Condori, Santos Daniel, Cardenas Fanny, Valverde Ada, Romero Soledad

**Affiliations:** HIV National Referential Laboratory, Instituto Nacional de Salud, Lima, Peru

## Abstract

**Introduction**: HIV drug resistance in Peru has been previously studied; however, data from general population of different regions of Peru is required. In this cohort, we have analyzed and described the resistance profile of HIV-infected Peruvian individuals sampled during period of 2008 and 2011.

**Materials and methods**: For this proposal, we have selected 297 patients corresponding to the ten most prevalent regions of Peru. To detect resistant mutation, we have used the Trugene commercial kit and an in-house Genotyping test. All patients included in this study were selected after of the approval of HIV Expert Committee of the Peruvian Ministry of Health.

**Results**: From 297 genotyped patients, 245 (82%) were resistant to one or more antiretroviral (ART). M184V was the most frequent resistant mutation (30% for children and 26% for adults). Resistant profile revealed that children showed more resistance to 3TC/FTC (78%), while in adults it was EFV/NVP (50%). Regarding of pan-sensible patients, they showed similar virological and immunological failure than resistant patients (CD4/µL <200 and viral load up to 5 log). Of interest, we have identified six patients showing resistance to every known antiretroviral drug (Pan-resistant virus), including a seven-year old subject. Additional sequence analysis revealed two HIV samples with unusual mutations (hypermutation) and multiple stop codons. Despite this fact, blood samples showed high viral load and low CD4 cell count. Finally, we found that from 24 resistant patients genotyped twice or more, only 15 (63%) received a change of the treatment scheme. However, only five of them (21%) experimented a decreasing of their viral load.

**Conclusions**: We showed that HIV resistance profile in Peru is complex and follows different molecular evolution depending of infected age-group. Moreover, our data suggest that a re-planning of therapy strategies should be performed to diminish the resistance in Peruvian population according of new resistant mutations and their resistance profile.

# Sub article: P20: Perinatal HIV transmission still occurs where HAART, perinatal care and safe alternative to breastfeeding do still exist

**DOI:** 10.7448/IAS.17.2.19124

**Published:** 2014-05-07

**Authors:** Foradori Irene, Stankievich Erica, Ivalo Silvina, Hakim Alejandro, Losso Marcelo, Burgoa Patricia, Adissi Lelia, Fernandez Marta

**Affiliations:** 1 Pediatrics–HIV, Hospital JM Ramos Mejia, Ciudad Autonoma de Buenos Aires, Argentina; 2 Infectology, Hospital JM Ramos Mejia, Ciudad Autonoma de Buenos Aires, Argentina; 3 Gynecology, Hospital JM Ramos Mejia, Ciudad Autonoma de Buenos Aires, Argentina; 4 Inmunology, Hospital JM Ramos Mejia, Ciudad Autonoma de Buenos Aires, Argentina; 5 Psychology, Hospital JM Ramos Mejia, Ciudad Autonoma de Buenos Aires, Argentina; 6 Social Services, Hospital JM Ramos Mejia, Ciudad Autonoma de Buenos Aires, Argentina

## Abstract

**Introduction**: Since 2003, the interventions to prevent the HIV mother-to-child transmission (MTCT) have been intensified and implemented in our hospital. However, risks associated with perinatal transmissions; namely poor prenatal care, late HIV diagnosis and lack or inadequate uses of antiretrovirals (ARVs) during pregnancy are still resulting in newborn infected babies. Our aim was directed to analyze the impact of different strategies to try to reduce MTCT and to describe relevant maternal and infant outcomes.

**Methods**: Observational, prospective and longitudinal study of a cohort of HIV-infected pregnant women admitted to a public hospital in Buenos Aires from January 2003 to December 2013.

**Results**: Three hundred and fifty-one deliveries attended to at this hospital resulted in 16 infected children, with an overall MTCT rate (TR) of 4.8%. Even when the TR during 2003/2007 dropped compared with the period 2008/2013: 4.2% vs. 3.1%, three babies became infected in 2013 (TR: 8.5%). Ninety three percent of mothers acquired HIV infection through unprotected sexual intercourse, and 70% were diagnosed before the current pregnancy. Eighty four percent had <10 years of formal education, and 26% were drug users or smoked tobacco. As high as 85.5% received ARVs during pregnancy, and 87.5% had HIV-RNA near to delivery <1000 cps/ml. Two transmissions were documented from mothers with HIV-RNA <1000 cps/ml at delivery. All cases of infected babies shared high risk characteristics for transmission as previously mentioned, including seroconversion during pregnancy. There was one neonatal death and one major congenital abnormality. Four children infected during this period were lost to follow up.

**Conclusions**: Our findings show the limitations of current interventions and healthcare systems to achieve the expected rates of HIV-MTCT. In this setting, periodic review of applied strategies, improved access for high risk cases and novel interventions are urgently needed to control and eliminate the persistent paediatric infections in settings as such, where the transmission “zero” is feasible.

# Sub article: P21: The influence of physical well-being, family and social support on the psychological and emotional quality of life dimensions of young people with HIV

**DOI:** 10.7448/IAS.17.2.19127

**Published:** 2014-05-07

**Authors:** dos Santos Mendes Mónico Lisete, Nobre Lima Luiza, Arraiol Diana, Araújo Rodrigues Francisco Rafael, Meireles Cardeira Hélder

**Affiliations:** 1 Faculty of Psychology and Education Sciences, University of Coimbra, Coimbra, Portugal; 2 Education, University of Coimbra, Development and Counselling, Coimbra, Portugal; 3 Institute of Biomedical Sciences Abel Salazar, University of Porto, Porto, Portugal; 4 Clinical Psychology, University of Extremadura, Extremadura, Spain

## Abstract

**Introduction**: The evaluation of health-related quality of life in children with chronic diseases has gained increasing interest as they face a host of psychological and social problems that need to be considered along with their physical treatments [1, 2]. The aim of this research is to analyze the influence of physical well-being, family and social support of children and adolescents with HIV on their self-perception, psychological well-being and emotions.

**Materials and methods**: Participants included 15 children and adolescents (8 to 17 years old; 11 boys; M=11.73 years old; SD=3.43) with HIV, recruited from the Consultation of Infectious Diseases of a Portuguese Pediatric Hospital. They responded to six dimensions of the Portuguese version of the KIDSCREEN-52 (The Kidscreen Group Europe, 2006; consistency coefficient of 0.821).

**Results**: The mean scores (from 0 to 100%) showed higher values for social support and peers (M=90.8; SD=15.3), followed by moods and emotions (M=84.7; SD=16.7), psychological well-being (M=84.6; SD=15.4), relations with parents and home life (M=82.9; SD=14.5), physical well-being (M=70.1; SD=33.3) and self-perception (M=57.5; SD=13.7). Altogether, relations with parents and home life, physical well-being and social support and peers explained 40% of the children and adolescents’ psychological well-being, 46% of their moods and emotions, but only 5% of their self-perception (see results of multivariate multiple regression in Figure 1). The relations with parents and home life did not show any significant influence. Inversely, the physical well-being had a strong influence on moods and emotions (β=0.59) and psychological well-being (β=0.46), but less in self-perception (β=0.22). Social support and peers showed a positive albeit moderate influence in psychological well-being (β=0.25) and moods and emotions (β=0.28), but not in self-perception.

**Abstract P21–Figure 1:** Multivariate Multiple Regression Analysis.

Multivariate Multiple Regression Analysis.

**Conclusions**: When considering the emotional and psychological health of young people with HIV, accounting for their physical well-being and supporting their socio-developmental tasks can serve as important protective factors, enhancing their quality of life and promoting more adaptive developmental pathways.

## References

[1] Malee K, Tassiopoulos K, Huo Y, Siberry G, Williams PL, Hazra R (2011). Mental health functioning among children and adolescents with perinatal HIV infection and perinatal HIV exposure. AIDS Care.

[2] Wang B, Li X, Barnett D, Zhao G, Zhao J, Stanton B (2012). Risk and protective factors for depression symptoms among children affected by HIV/AIDS in rural China: a structural equation modeling analysis. Soc Sci Med.

# Sub article: P22: The emotional burden and quality of life of informal caregivers of children and adolescents with HIV

**DOI:** 10.7448/IAS.17.2.19131

**Published:** 2014-05-07

**Authors:** Nobre Lima Luiza, dos Santos Mendes Mónico Lisete, Monteiro Domingues Lorena, Araújo Rodrigues Francisco Rafael, Meireles Cardeira Hélder

**Affiliations:** 1 Faculty of Psychology and Education Sciences, University of Coimbra, Coimbra, Portugal; 2 Education, Development and Counselling, University of Coimbra, Coimbra, Portugal; 3 Institute of Biomedical Sciences Abel Salazar, University of Porto, Porto, Portugal; 4 Clinical Psychology, University of Extremadura, Extremadura, Spain

## Abstract

**Introduction**: The care giving experience has an impact on every facet of a caregiver's life, from physical to psychological and emotional health [1]. Caregivers of children with chronic diseases face circumstances that challenge their adaptation to the disease and influence their well-being. The aim of this research was to study the role emotional burden plays in predicting the quality of life of informal caregivers of children infected with HIV.

**Materials and methods**: The sample comprised 20 Portuguese informal caregivers (17 women; M=42.7 years old, DP=10.2) of 20 children and adolescents with HIV (6 to 17 years old). Subjects answered to the Portuguese version of The Revised Burden Measure (RBM; Montgomery et al., 2006) composed by four dimensions—Burden of the relationship (Cronbach's α=0.86; M=10.1; SD=7.2), Objective burden (α=0.89; M=16.2; SD=7.2), Subjective burden (α=0.93; M=14.8; SD=6.8) and Personal growth (α=0.77; M=22.0; SD=5.3)—and to the World Health Organization Quality of Life Questionnaire [2] that measures four quality of life domains—Physical health (α=0.89; M=65.2; SD=23.3), Psychological health (α=0.91; M=67.5; SD=22.2), Social relationships (α=0.87; M=64.6; SD=24.2) and Environment (α=0.87; M=60.2; SD=19.9).

**Results**: The four dimensions of emotional burden explain 53.4% of the caregiver's quality of life, Rmultiple=0.73, F(4,15)=4.3, *p*<0.05, with the Psychological health domain being the most affected (R^2^=0.51), followed by Environment (R^2^=0.36), Physical health (R^2^=0.34) and Social relationships (R^2^=0.33). The three types of emotional burden predict negatively on the four domains of quality of life, whereas Personal growth predicts positively on Psychological health, though with low magnitude (see Figure 1). The Subjective burden is the strongest predictive dimension of the four quality of life domains.

**Abstract P22–Figure 1:** Multivariate multiple regression of the quality of life (WHQOL) predicted by the emotional burden.

Multivariate multiple regression of the quality of life (WHQOL) predicted by the emotional burden.

**Conclusions**: Since subjective burden seems to be the main negative predictor of caregivers’ physical and psychological quality of life, it might be important that social and health services develop adequate responses in considering caregivers’ welfare when the well-being of children and adolescents with HIV is at stake.

## References

[1] Dyck G, Short R, Vitalino P (1999). Predictors of burden and infectious illness in schizophrenia caregivers. Psychosom Med.

[2] Fleck MP, Louzada S, Xavier M, Chachamovich E, Vieira G, Santos L (2000). Aplicaçäo da versäo em português do instrumento WHOQOL-bref. Rev Saúde Pública.

[3] Lindag M, Zerden M, Ferrando S, Testa M (2011). HIV+ caregivers and HIV+ non-caregivers: differences in sociodemographics, immune functioning, and quality-of-life. Aids Care.

# Sub article: P23: Elvitegravir/cobicistat/emtricitabine/tenofovir DF demonstrates durable efficacy and safety versus efavirenz/emtricitabine/tenofovir DF at Week 144

**DOI:** 10.7448/IAS.17.2.19132

**Published:** 2014-05-07

**Authors:** Hawkins Trevor, Campo Rafael, Wohl David, Cohen Cal, Gallant Joel, Mills Anthony, Sax Paul, Zolopa Andrew, Hui C Liu, Kirsten L White, Martin S Rhee, Szwarcberg Javier

**Affiliations:** 1 Infectious Diseases, Southwest Care Center, Santa Fe, NM, USA; 2 University of Miami, Miller School of Medicine, Infectious Diseases, Miami, FL, USA; 3 University of North Carolina, Infectious Diseases, Chapel Hill, NC, USA; 4 Community Research Initiative of New England, Clinical Research HIV, Boston, MA, USA; 5 Anthony Mills MD, Inc, Infectious Diseases/HIV, Los Angeles, CA, USA; 6 Brigham and Women's Hospital, Harvard Medical School, Infectious Diseases, Boston, MA, USA; 7 Clinical Research, Stanford University, Palo Alto, CA, USA; 8 Programing, Gilead Sciences, Foster City, CA, USA; 9 Gilead Sciences, Clinical Virology, Foster City, CA, USA; 10 Clinical Research HIV, Gilead Sciences, Foster City, CA, USA

## Abstract

**Introduction**: In this randomized, double-blind controlled Phase 3 trial in treatment-naïve patients, elvitegravir/cobicistat/emtricitabine/tenofovir DF (STB) was non-inferior to efavirenz/emtricitabine/tenofovir DF (ATR) at Wk 48 with durable efficacy and favourable safety through Wk 96. We report Wk 144 data.

**Methods**: Eligibility criteria included HIV-1 RNA (VL) ≥5000 c/ml and eGFR ≥70 ml/min. Virologic success (VL <50 c/ml) was assessed per snapshot algorithm.

**Results**: A total of 700 patients were randomized and treated. Through Wk 144, high rates of virologic success were maintained (STB 80% vs. ATR 75%, difference 4.9%, 95% CI −1.3% to 11.1%). Mean CD4 cell increase was 321 vs. 300 cells/µL. Virologic success was similar between groups in patients with baseline VL >100,000 c/ml (77% vs. 78%) and those with CD4 ≤350 cells/µL (76% vs. 76%). Emergent resistance was infrequent (3% vs. 4%); no STB patient developed resistance after Wk 96. Drug discontinuation due to adverse events (AEs) was low and similar (6% vs. 7%); of those, four vs. two patients discontinued after Wk 96 due to an AE. Renal discontinuation occurred in eight (2.3%) vs. zero patients with no cases of proximal tubulopathy after Wk 24. Neuropsychiatric AEs were lower with STB (51% vs. 68%, *p*<0.001), as was rash (25% vs. 32%, *p*=0.044). Median changes in creatinine (mg/dl) at Wk 144 (0.14 vs. 0.01) were similar to those at Wk 48. STB was associated with smaller median increases (mg/dl) in total (16 vs. 20, *p*=0.007) and LDL cholesterol (12 vs. 18, *p*=0.007) and similar increase in triglycerides (2 vs. 2); changes in total cholesterol to HDL ratio were similar (−0.3 vs. −0.3).

**Conclusions**: At Wk 144, STB demonstrated high rates of virologic suppression regardless of baseline viral load and CD4 cells, low rates of resistance and a favourable safety profile with no new renal safety signals. These results support the durable efficacy and long-term safety of STB.

# Sub article: P24: Long-term efficacy and safety of elvitegravir/cobicistat/emtricitabine/tenofovir DF versus atazanavir plus ritonavir plus emtricitabine/tenofovir

**DOI:** 10.7448/IAS.17.2.19134

**Published:** 2014-05-07

**Authors:** Clumeck Nathan, Molina Jean-Michel, Henry Keith, Gathe Joe, Jürgen H Rockstroh, DeJesus Edwin, Wei Xuelian, Kirsten L White, Marshall W Fordyce, Martin S Rhee, Szwarcberg Javier

**Affiliations:** 1 Saint Pierre University Hospital, Infectious Diseases, Brussels, Belgium; 2 Hopital Saint-Louis, AP-HP and University of Paris Diderot, Infectious Diseases, Paris, France; 3 Hennepin County Medical Center, Infectious Diseases, Minneapolis, MN, USA; 4 Therapeutic Concepts, P.A., Clinical Research HIV, Houston, TX, USA; 5 Research, Universitätsklinikum Bonn, Bonn, Germany; 6 Orlando Immunology Center, Clinical Research, Orlando, FL, USA; 7 Biostatistics, Gilead Sciences, Foster City, CA, USA; 8 Clinical Virology, Gilead Sciences, Foster City, CA, USA; 9 Clinical Research HIV, Gilead Sciences, Foster City, CA, USA

## Abstract

**Objective**: In this randomized, double-blind, active-controlled Phase 3 trial in treatment naïve patients, elvitegravir/cobicistat/emtricitabine/tenofovir DF (STB) was non-inferior to atazanavir boosted by ritonavir (ATV+RTV)+emtricitabine/tenofovir DF (FTC/TDF) at Week 48 with durable efficacy and a favourable safety profile through Week 96. We report Week 144 data.

**Methods**: Key eligibility criteria included HIV-1 RNA ≥5000 c/ml and eGFR ≥70 ml/min. Virologic success (HIV-1 RNA <50 c/ml) was assessed per snapshot algorithm. Bone mineral density (BMD) was assessed in a sub-study.

**Results**: A total of 708 patients were randomized and treated. Through Week 144, high rates of virologic success were maintained (STB 78% vs. ATV+RTV+TVD 75%, difference 3.1%, 95% CI −3.2% to 9.4%). Virologic success was similar in patients with HIV-1 RNA >100,000 c/ml (75% vs. 72%) and those with CD ≤350 cells/µL (76% vs. 74%). Mean (±SD) CD4 cell increases (cells/mm3) were +280 (±159.8) vs. +293 (±211.5). Emergent resistance was infrequent in both groups (2% vs. 1%). Drug discontinuation due to adverse events (AEs) was low and comparable (6% vs. 8%). Renal discontinuation occurred in 5 (1%) vs. 8 (2%) patients; of those, two vs. six patients discontinued after Week 96, including three ATV+RTV+TVD patients with proximal renal tubulopathy (PRT). No cases of PRT occurred in STB group. Mean changes from baseline in creatinine (µmol/L [mg/dl]) at Week 144 were 10.6 vs. 7.1 [0.12 vs. 0.08] and were stable since Week 48. STB had smaller mean decreases (%) in BMD (hip: −2.83 vs. −3.77, *p*=0.23 spine: −1.43 vs. −3.68, *p*=0.018).

**Conclusions**: At Week 144, STB, the only INI-based single-tablet regimen for HIV, demonstrated high rates of virologic suppression regardless of baseline viral load and CD4 cells, with low rates of resistance and a favourable safety profile with no new renal safety signals. These results support the durable efficacy and long-term safety of STB.

# Sub article: P26: Cardiovascular risk in HIV-infected patients in one cohort in Mexico

**DOI:** 10.7448/IAS.17.2.19166

**Published:** 2014-05-07

**Authors:** Ruiz Herrera Vida, Crabtree Ramirez Brenda, Caro-Vega Yanink, Sierra-Madero Juan

**Affiliations:** Department of Infectious Diseases, Instituto Nacional de Ciencias Médicas y Nutrición Salvador Zubirán, Mexico City, Mexico

## Abstract

**Introduction**: Cardiovascular disease (CVD) is currently a leading cause of morbidity and mortality in HIV patients [1]. This is probably a result of combination of an increased prevalence of risk factors (RFs) exposure to ART and a direct effect of HIV. The Framingham Risk Score (FRS) is a calculated measure of CVD risk, recommended for routine HIV care. Identifying CVD risk scores in a given population is important to plan for interventions and future strategies [3]. Little information on CVD risk in HIV-infected patients has been reported from Latin America. The aim of this study was to determine the CVD risk profile in a large well-characterized Mexican cohort.

**Materials and methods**: Cross-sectional study of HIV-infected adults in the HIV clinic at INCMNSZ (in Mexico City) in the year 2013. CVD risks were calculated using the 10-year risk sex-specific Framingham equations, which was then categorized in high risk: Equivalent to CVD >20% and/or DM, intermediate risk: 10%–20% and low risk: <10% [2]. Subgroups were compared using Chi-squared or Fishers exact test for categorical variables and Mann–Whitney test for continuous variables. For statistical analysis, the STATA V11 was used.

**Results**: A total of 1453 patients formed the complete cohort of whom 1096 had complete information. Nine hundred and fifty-nine (87.5%) of 1096 patients analyzed were men, the median age was 41 (IQR 34–49). CVD was present in 22 (2%). Diabetes was present in 81 patients (7.39%), hypertension in 47 (4.29%) and Current smokers were 256 (23.36%). A total of 1044 (95.26%) were on ART, of whom 908(85%) had viral load <40 copies/ml. The median duration of c-ARV was 56 (IQR 34–105) (months), c-HDL was 41 (IQR 35–48) (mg/dl), total cholesterol (TC) 177 (IQR 152–206) (mg/dl) and triglycerides 162 (IQR 114–232) (mg/dl). Body mass index (BMI) was >25 in 381 (37%) and 114 (11.1%) had a BMI >30. Estimated 10-year CHD risk was >20% in 98 patients (8.9%), 10%–20% in 61 (5.56%) and 937 (85.5%) <10%.

**Abstract P26–Table 1:** Clinical and demographic characteristics stratified by Framingham Risk Score

*N*=1096 (%)	Low risk (<10%) *N*=937 (85.49%)	Intermediate risk (10-20%) *N*=61 (5.56%)	High risk (>20%) *N*=98 (8.94%)	p*
Age (y) (IQR)	39 (33-46)	56 (49–62)	52.5 (46–62)	0.000
Gender: Male	815	61	83	0.000
Median triglyceride (mg/dl) (IQR)	157 (110–218)	218 (150–316)	213 (139–339)	0.000
Current smoker	199 (21.2)	30 (49.1)	27 (27.6)	0.000
Hypertension, male/female	18/5	5/0	15/4	0.000
Diabetes mellitus, male/female	0	0	66/15	0.000
Median duration of cARV (months) (IQR)	54.5 (33–97)	90.5 (44.2–151)	68 (42.8–171)	0.020
BMI (>30)	103 (11.8)	2 (3.2)	9 (10.1)	0.404
CD4 count (cells/ul)	477 (336–651)	536 (378–769)	471 (349–628)	0.317
HIV RNA >40 copies/ml (detectable)	120	2	10	0.070
History of CV event	11 (1.17)	3 (4.92)	8 (8.16)	0.000

IQR: Interquartile range (25th percentile, 75th percentile), m: months, y: years, cARV: combination antiretroviral therapy. p*: *p*-value *χ: Chi-square test for categorical variables and U-Mann–Whitney test for continuous variables.

Clinical and demographic characteristics stratified by Framingham Risk Score

IQR: Interquartile range (25th percentile, 75th percentile), m: months, y: years, cARV: combination antiretroviral therapy. p*: *p*-value *χ: Chi-square test for categorical variables and U-Mann–Whitney test for continuous variables.

**Conclusions**: This cohort of HIV-infected subjects in Mexico has a low proportion of high CVD risk individuals, mostly given by diabetes. CVD risk assessment in HIV-infected patients can help identify patients that may benefit from interventions including novel ARV strategies associated with lower risk.

## References

[1] Thienemann F, Sliwa K, Rockstroh JK (2013). HIV and the heart: the impact of antiretroviral therapy: a global perspective. Eur Heart J.

[2] Currier JS (2009). Update on cardiovascular complications in HIV infection. Top HIV Med.

[3] Bavinger C, Bendavid E, Niehaus K, Olshen RA, Olkin I, Sundaram V PLoS ONE.

# Sub article: P27: Relationship between depressive symptoms and health-related quality of life in Mexican HIV-positive patients: a multiple linear regression model

**DOI:** 10.7448/IAS.17.2.19167

**Published:** 2014-05-07

**Authors:** Rodríguez Victor, Vega Hamid, Hernández Gabriela, Cruz Jeremy

**Affiliations:** Mental Health, Condesa Specialized Clinic, Mexico City, Mexico

## Abstract

**Introduction**: International studies estimate the prevalence of depressive symptoms in HIV patients between 15% and 44%. Additionally, 75% of patients report that at some stage of the disease, they could not perform their daily activities [decreased quality of life (QoL)] due to the HIV-associated fatigue or depressive symptoms. The Medical Outcomes Study Short Form-36 (SF-36) is the most useful questionnaire for assessment of Health Related QoL (functional state), and Beck Depression Inventory (BDI) is the most useful instrument for assessment of depressive symptoms in clinic populations. Previously, these two instruments were validated in a sample of Mexican people with HIV 1, 2 and 3. Because of the high prevalence of depression in this population, the degree of associated dysfunction and the little updated research in this field in Mexico, we aimed to determine the relationship between depression and QoL with previously validated instruments in patients with HIV.

**Materials and methods**: This cross-sectional study included a non-probabilistic sample of patients that completed the locally adapted and validated BDI and Mexican SF-36. The questionnaires were applied with the regular mental health assessment at the Condesa Clinic (2012–2013), but we only considered patients under antiretroviral treatment. The statistical analysis was performed with SPSS v. 20. A *p*<0.01 was considered to be significant.

**Results**: Total sample was 380 (84% male. Mean age was 37.8±9.1 years old, 11.5±3.4 years of education and 6.9±5.4 years after diagnosis of HIV. The average score of the SF-36 was 68.84±17.83 (compared to 89.95±6.14 in general Mexican population) and 11±10 in the BDI (mild to moderate depression). The BDI score had significant correlation with seven of eight domains of SF-36 (*r*=0.330–0.597). The strongest correlations were with the domains of vitality, mental health, social functioning and total score (*r*>0.500). We found higher SF-36 scores in patients with <6.9 years from diagnosis of HIV (*t*=2.267). After multiple linear regression model, depressive symptoms explained 33% of the variance (adjusted R2) of QoL, taking SF-36 total score as the main outcome.

**Conclusions**: This study shows the negative impact in daily life of HIV patients, even if they were on antiretroviral treatment, demonstrated with the 20% less of SF-36 total score than general population. Depressive symptoms explain a significant percent of the variance of the functional status (QoL) of HIV patients, suggesting that treating depressive symptoms with appropriate antiretroviral therapy may reduce the impact on the patient's functionality and aids reintegration into daily life.

## References

[1] Gaynes BN, Pence BW, Eron JJ, Miller WC (2008). Prevalence and comorbidity of psychiatric diagnoses based on reference standard in an HIV+ patient population. Psychosom Med.

[2] Rodriguez V, Magis C, Casillas J, Vega Ramirez H, Hernandez G, Ramirez Renteria C Internal consistency and factorial structure of the Depression Beck Inventory (BDI) in Mexican HIV-positive patients.

[3] Rodriguez V, Vega H, Hernandez G, Ramirez C, Cruz J, Casillas J Reliability, validity and factorial congruence of SF-36 Health Survey in Mexican HIV-positive patients.

# Sub article: P28: Characteristics of Truvada^®^ for pre-exposure prophylaxis users in the United States

**DOI:** 10.7448/IAS.17.2.19168

**Published:** 2014-05-07

**Authors:** Silva Anita, Mera Robertino, Ng Leslie, Magnuson David, Rawlings Keith

**Affiliations:** 1 Public Health & Medical Affairs, Gilead Sciences, Sao Paulo, Brazil; 2 Epidemiology, Gilead Sciences, Foster City, CA, USA; 3 Drug Safety and Public Health, Gilead Sciences, Foster City, CA, USA; 4 Medical Affairs, Gilead Sciences, Foster City, CA, USA

## Abstract

**Introduction**: Truvada^®^ (TVD) was approved in July 2012 by the U.S. FDA for the pre-exposure prophylaxis (PrEP) in combination with safer sex practices to reduce the risk of sexually acquired HIV-1 in adults at high risk. Registration studies in the United States accrued only males while serodiscordant couple studies were carried out in Africa. The objective of this study is to explore the characteristics of the U.S. PrEP population and their prescribers.

**Materials and methods**: A standard algorithm was used to identify TVD for PrEP by excluding use for HIV treatment, post-exposure prophylaxis and off-label treatment for chronic hepatitis B. National electronic patient level data from ~55% of all U.S. retail pharmacies that dispensed TVD between January 2012 and September 2013 was used. De-identified patient-level data including detailed drug information, medical claims and patient demographics were analyzed. Logistic regression was used to estimate the odds of change over time.

**Results**: A total of 2319 unique individuals who started TVD for PrEP after 1 January 2012 were included in the analysis. As high as 48.8% of PrEP users were women. Mean age was 38.2+12.2 years, with males being significantly older (39.5+12.0) than females (36.8+12.3) and 12.3% of individuals were under 25 years old. Proportion of males under 25 was 8.0%, 95% CI 6.5–9.5; significantly lower than that of women, 16.8%, 95% CI 14.6–18.9. Distribution of TVD for PrEP prescriptions by specialty: family practice (18%), internal medicine (16%), infectious diseases (11%), nurse practitioners and physician assistants (9%) each. Among women who initiated TVD for PrEP, 49% received it from nurse practitioners and 43% infectious diseases specialists. When compared to HIV positive patients, uninfected individuals receiving TVD for PrEP were 1.8 times more likely to be female (95% CI 1.6–1.9), 1.4 times more likely to be younger than 25 years old (95% CI 1.3–1.7) and 3.6 times more likely to be treated by a non-ID physician (95% CI 3.2–4.1).

**Conclusions**: The population of TVD for PrEP users in the United States is quite different from HIV+ subjects. They are more likely to be women, younger and be treated by Primary care clinicians.

# Sub article: P29: Week 144 resistance analyses of the phase 3 EVG/COBI/FTC/TDF studies

**DOI:** 10.7448/IAS.17.2.19169

**Published:** 2014-05-07

**Authors:** Kulkarni Rima, Michael E Abram, Martin S Rhee, Marshall W Fordyce, Szwarcberg Javier, Michael D Miller, Kirsten L White

**Affiliations:** 1 Research and Development, Gilead Sciences, Inc., Foster City, CA, USA; 2 Clinical Research HIV, Gilead Sciences, Inc., Foster City, CA, USA

## Abstract

**Introduction**: Two phase 3, randomized, blinded studies of elvitegravir/cobicistat/emtricitabine/tenofovir DF (EVG/COBI/FTC/TDF) in treatment-naïve subjects are ongoing (Studies 102 and 103). Virologic responses were EVG/COBI/FTC/TDF vs. efavirenz ([EFV]/FTC/TDF) (82% vs. 78%) and EVG/COBI/FTC/TDF vs. ritonavir-boosted atazanavir (ATV+RTV)+FTC/TDF (81% vs. 79%) at W144. Resistance analyses through W144 are presented.

**Materials and methods**: HIV-1 genotypes (PR and RT) were analyzed at screening. Retrospective integrase (IN) genotyping was conducted on 363 EVG/COBI/FTC/TDF baseline samples. Virologic failures had genotypic/phenotypic analyses at confirmed failure and baseline for PR, RT and IN, or at first failure (EVG/COBI/FTC/TDF only) (Monogram).

**Results**: EVG/COBI/FTC/TDF subjects with baseline PI (*N*=18) or NNRTI mutations (*N*=95) including K103N in RT (*N*=27, Study 103) had high virologic response through W144 (82% for K103N). Baseline primary IN strand transfer inhibitor (INSTI) mutations were rare (*N*=4/337); all EVG/COBI/FTC/TDF subjects with T97A (*N*=3) and Y143H (*N*=1) rapidly suppressed and had W144 HIV-1 RNA <50 c/ml. The EVG/COBI/FTC/TDF group through W144 had 18 subjects (2.6%; 18/701) that developed primary INSTI (*N*=15) and/or NRTI resistance mutations (*N*=17) (Any resistance: 13 through W48, 3 between W48 and W96, and 2 between W96 and W144). Emergent INSTI mutations were E92Q (*N*=9), N155H (*N*=5), Q148R (*N*=3), T66I (*N*=2) and T97A (*N*=1) and emergent NRTI mutations were M184V/I (*N*=17) and K65R (*N*=5). The EFV/FTC/TDF group had 14 subjects (4.0%; 14/352) that developed drug resistance and was most commonly K103N (*N*=13) with M184V/I (*N*=4) plus K65R (*N*=3) in RT. The ATV+RTV+FTC/TDF group had two subjects with M184V/I. First failure analysis found M184V/I develops first, and resistance was not found to NRTIs in 4/18 subjects and to INSTIs in 10/18 subjects.

**Conclusions**: Treatment with EVG/COBI/FTC/TDF achieved durable high rates of virologic suppression in HIV-1 treatment-naïve subjects, including those with pre-existing NNRTI or PI resistance. Resistance development was infrequent (2.6% of STB-treated subjects). The most common patterns of resistance mutations to EVG/COBI/FTC/TDF were E92Q, Q148R or N155H in IN with M184V/I in RT, which were more readily detected at the confirmed virologic failure time point.

# Sub article: P30: Investigation of HIV-1 drug-resistant variants in plasma bulk and cell-associated strains ultra-deep sequencing from blood donors in Recife, Brazil

**DOI:** 10.7448/IAS.17.2.19170

**Published:** 2014-05-07

**Authors:** Pessôa Rodrigo, Felix Alvina Clara, Loureiro Paula, Sabino Ester Cerdeira, Michael P Busch, Sanabani Sabri Saeed

**Affiliations:** 1 Virology, Institute of Tropical Medicine, University of São Paulo, São Paulo, Brazil; 2 Hematologia e Hemoterapia, Fundação de Hematologia e Hemoterapia de Pernambuco–HEMOPE, Recife, Brazil; 3 Department of Infectious Disease, University of São Paulo, São Paulo, Brazil; 4 Blood Systems Research Institute, San Francisco, CA, USA

## Abstract

**Introduction**: This study aimed to better understand the resistance profile between plasma and peripheral blood mononuclear cells (PBMCs) compartments and quantify the prevalence of low-abundance drug-resistant viral variants in the proviral DNA. To this end, primary drug resistance mutations (PDRM) on viral DNA and plasma was performed in 24 blood donors from Recife, capital of Pernambuco in the north-east region of Brazil.

**Materials and methods**: Blood samples were obtained from 24 patients in whom PDRM from plasma had previously been identified using consensus bulk sequencing. For proviral DNA, amplicons were molecularly bar-coded, pooled and sequenced by Illumina paired-end protocol. The minority HIV-1 resistant variants were identified using a threshold of >1.0% of the sequenced reads. All amino acid positions associated with PDRM were identified according to standard methods.

**Results**: Discordant data between the plasma and PBMCs virus were found in 16 of 24 donors. Six of these strains displayed major amino acid changes only in the cellular compartment and four of which had detectable major amino acid changes at prevalence between 1% and 20% of the sequenced population. Neither major amino acid changes in the protease (PR) nor in the reverse transcriptase (RT) coding region were detectable by plasma bulk sequencing. The overall frequency of minor mutations in both compartments in the PR gene was significantly greater than that detected in RT. Eight and one resistant mutations in the PR and RT regions, respectively, were detected in plasma bulk sequencing but were not observed in deep sequencing (DS) data. For the PR gene, major PR PDRM, namely, V82I, M46I and L33F were detected in five blood donors. The proviral DNA DS analysis in the RT region showed major PDRM at the following codons in five patients: M184I, M230I and E138K.

**Conclusions**: Our results showed different resistance profile between plasma and PBMC compartments and illustrated that resistant strains may be present at low prevalence within the proviral DNA sequenced population. Consistent with other studies, our results indicated that standard bulk sequencing is not sensitive enough to detect all mutations archived in the proviral DNA. An implication of this is the possibility that low abundant resistant variants could be selected and emerges as major virus populations when therapy is started.

# Sub article: P33: Rilpivirine resistance mutations in patients failing first-line non-nucleoside reverse transcriptase inhibitor (NNRTI) treatments in Argentina

**DOI:** 10.7448/IAS.17.2.19173

**Published:** 2014-05-07

**Authors:** Kademian Silvia, Bissio Emiliano, Altamirano Natalia, Castro Gonzalo, Barbas M Gabriela, Juan F De Leon, Cudola Analia

**Affiliations:** 1 Molecular Biology, Central Laboratory, Ministry of Health Cordoba, Cordoba, Argentina; 2 Ministerio de Salud de Nacion, Buenos Aires, Argentina; 3 Biochemistry, Central Laboratory, Ministry of Health Cordoba, Cordoba, Argentina; 4 Quality Department, Central Laboratory, Ministry of Health Cordoba, Cordoba, Argentina; 5 Microbiology, Cordoba Catholic University, Cordoba, Argentina

## Abstract

**Introduction**: Following treatments based on NNRTIs, cross-resistance among efavirenz (EFV), etravirine (ETR) and/or nevirapine (NVP) is likely after virological failure, and could account as well for the development of resistance to rilpivirine (RPV). Actually, we reported a previous study regarding resistance emergence to ETR, the first second-line agent of this class [1]. RPV is the newest agent approved by the FDA for HIV-1 treatment of naïve adult patients. Additionally, it may be used in switching therapy from EFV, in virologically suppressed patients [2].

**Aim**: To evaluate the prevalence of resistance-associated mutations (RAMs) and sensitivity to RPV in antiretroviral experienced patients failing to other NNRTIs.

**Materials and methods**: A retrospective study was performed at the Public Health Central Laboratory in Córdoba, Argentina, over 210 samples received from August 2013 to January 2014, for routine HIV resistance testing. A total of 103 cases that showed resistance to one or more NNRTIs were selected for the present analysis. Samples were genotyped using Trugene System [3]. HIV Stanford Database Program was used as an HIV variant sub-typing tool [4].

**Results**: All the cases studied were resistant to NVP, whereas 73.8% were resistant to EFV. As for RPV, an overall reduced sensitivity of 35% was found, and for ETR, 63% of cases retained sensitivity to the drug. Prevalence of RAMs associated to RPV was as follows: Y 181C/I/V (18.4%), K101E/P (14.6%), H221Y (10.7%), Y188L/H/C (9.7%), E138A/K=L100I+K103N (5.8%), V179 D (4.9%) and M230L=F227 L (2.9%). As for HIV variants, 61% classified as B subtype and 39% as non-B subtype (being CRF_FB the most prevalent circulating recombinant form). There was no association between reduced sensitivity to RPV and virus subtype (38% B vs. 30% non-B, *p*=0.16, Fisher test.

**Conclusions**: Whilst natural polymorphisms could influence the barrier to drug resistance, the overall prevalence of resistance to RPV did not differ significantly when comparing B vs. non-B subtypes. Almost all RAMs related to RPV were relatively frequent among NNRTIs experienced subjects, suggesting the local circulation of viral strains with reduced sensitivity to the novel agent. This study points out the need for resistance testing to determine whether the emergence of RAMs associated to RPV rules out the use of NNRTIs in clinical practice.

## References

[1] Cudola A, Kademian S, Barbas M, DeLeon J Cross-resistance among non-nucleoside reverse transcriptase inhibitors (NNRTIs: Genotypic evaluation of the sensitivity to Etravirine (ETR)in HIV-1 infected patients following failure to first line treatments based on Nevirapine(NVP) and/or Efavirenz(EFV).

[2] Sharma M, Saravolatz LD (2013). Rilpivirine: a new non-nucleoside reverse transcriptase inhibitor(Review). J Antimicrob Chemother.

[3] HIV-1 Trugene Genotyping kit, for use with the DNA OpenGene™ system, Siemens Healthcare Diagnostics

[4] HIVdb Program Genotype Resistance Interpretation, 6.3.1 Version (update 09/10/13). http://hivdb.stanford.edu.

# Sub article: P34: Genotypic frequency of mutations to antiretroviral in patients with HIV in Venezuela during the period 2006–2012

**DOI:** 10.7448/IAS.17.2.19174

**Published:** 2014-05-07

**Authors:** Ameli Gladys, Gutíerrez Cristina, D'Angelo Pierina, Aguilar Marwan, Molina Moraima, Carballo Martin, Hernández Marbelys, Comegna Mario, Roldan Yajaira, Rosales Anselmo, Lopez Maria Graciela, Deibis Leopoldo, Morales Miguel, Napoleón Guevara Rafael

**Affiliations:** 1 Virology, National Institute of Hygiene Rafael Rangel, Caracas, Venezuela; 2 Virology, National Committee of Resistance, Caracas, Venezuela; 3 University Central, Caracas, Venezuela

## Abstract

**Introduction**: The emergence of mutations associated with resistance to antiretroviral drugs (ARV) HIV-1 limits the effectiveness of treatment in HIV-infected patients, leading to virological and immunological failure. Secondary resistance occurs in individuals receiving treatment due to mutations of the virus and selection of resistant variants during exposure to ARVs. The aim of this study is to describe the most frequent mutations in the reverse transcriptase region and HIV protease associated with secondary resistance to ARVs.

**Materials and methods**: A total of 823 plasma samples from HIV-infected with virologic failure receiving ARV treatment patients, who were referred to the National Institute of Hygiene “Rafael Rangel” in Caracas, Venezuela, during the period from June 2006 were studied to December 2012 by CONARESAR. Each sample was processed for plasma viral load determination and test of resistance by HIV genotyping Trugene Kit^®^.

**Results**: Of the samples tested, in 97 (12%) patients, no resistance mutations were detected, while 87% of the isolates showed secondary resistance mutations, of which 72% had mutations in both regions of reverse transcriptase and protease and 28% of patients had resistance mutations in either of the two regions studied. According to the group of ARV drugs, the most common resistance mutations associated with nucleoside reverse transcriptase inhibitors (NRTIs) were M184I/V (20%), T215C/F/S/V/Y (13%), D67N (11%), M41L (9%), K219Q/E (8%) and L74V (7%), and the most common resistance mutations associated with non-nucleoside inhibitors of reverse transcriptase inhibitors (NNRTIs) were K103H/N (40%), L100I (11%) K101E/P/Q (8%) and G190A/E/S (5%). Finally, mutations to the protease inhibitors (PI) were M36I/L/V (19%), A71V/T (18%), I54A/L/M/S/T/V (16%), M46I/L (15%) and L90M (14%).

**Conclusions**: In Venezuela, like other studies previously reported found a high level of secondary resistance to antiretrovirals, predominantly found in the NRTI mutations, followed by those associated with NNRTI and IP. These results also suggest studies of primary resistance in the country are necessary to assess the possible transmission of resistant strains due to the high frequency of resistance mutations found in our study.

# Sub article: P35: Resistance mutations and polymorphisms in patients infected with HIV-1, treated with raltegravir in Venezuela

**DOI:** 10.7448/IAS.17.2.19175

**Published:** 2014-05-07

**Authors:** Ríos Maricruz, Gutiérrez Cristina, Ameli Gladys, D′Angelo Pierina, Hernández Marbelys, Rangel Héctor, Garzaro Domingo, Pujol Flor, Napoleón Guevara Rafael

**Affiliations:** 1 Virology, National Institute of Hygiene, Caracas, Venezuela; 2 Programa ITS/SIDA, Ministerio del Poder Popular para la salud, Caracas, Venezuela; 3 Microbiología, Instituto venezolano de Investigaciones Cientificas (IVIC), Caracas, Venezuela; 4 University Central, Caracas, Venezuela

## Abstract

**Introduction**: There are few antiretrovirals that inhibit the action of HIV-1 integrase. Some treatments fail to produce mutations in this region; however, the majority of patients under antiretroviral therapy have shown good adhesion and is therefore very limited experience in patients with suspected virologic failure in this country. The aim of this study was to investigate the presence of resistance mutations and polymorphisms in the integrase coding region in the Venezuelan population infected with HIV-1 under antiretroviral therapy with integrase-inhibitors.

**Materials and methods**: Plasma samples from six patients were studied under treatment with raltegravir, with suspected virologic failure, prior informed consent of the same, by a technique of chain reaction polymerase with specific primers that amplify the region between the codons 7–288 of the pol gene. The PCR products were sequenced and analyzed by database Stanford University.

**Results**: The presence of resistance mutations G140S and Q148H was detected in 33% (2/6) of the patients, and only in 16% (1/6) of these, the Y143H mutation pattern was observed. Such patterns have been reported like primary resistance mutation and G140S as a compensatory resistance mutation. In the rest of the samples, some polymorphisms not associated with resistance to these inhibitors were observed.

**Conclusions**: The results represent the first report of the presence of mutation associated with resistance to integrase inhibitors of HIV-1 in the Venezuela population. However, the number of samples tested was very low because there are few cases with suspected failure of adherence to treatment and those who are under this antiretroviral therapy have remained with undetectable viral loads to date.

# Sub article: P36: Resistance mutations profile of antiretroviral-experienced Colombian adults infected with HIV-1, 2010–2013: a cross-sectional study

**DOI:** 10.7448/IAS.17.2.19176

**Published:** 2014-05-07

**Authors:** Perez Luz Eugenia, Monsalve Maria Alejandra, Montufar Maria Camila, Naranjo Luisa Fernanda, Gonzalez Andres, Gonzalez Juan Carlos

**Affiliations:** 1 School of Medicine, Universidad Pontificia Bolivariana, Medellin, Colombia; 2 Molecular Biology Laboratory, Dinamica IPS, Medellin, Colombia; 3 Scientific Direction, Clinica Cardio VID, Medellin, Colombia

## Abstract

**Introduction**: The aim of this study is to describe the resistance mutation profile of 204 HIV-1-infected Colombian patients undergoing genotypic assays testing.

**Materials and methods**: A retrospective, reference laboratory-based study was performed between October 2010 and June 2013. All the adult patients with HIV-1 infection tested for genotyping to rule out therapeutic failure were included in the analysis. All the patients were receiving first-line antiretroviral therapy (ART). Genotypic resistance assays were performed using TRUGENE HIV-1 genotyping kit assay and the Gene Objects 4.1 Software (Siemens Healthcare Diagnostics) for the detection of mutations in the HIV-1 protease (PR) and reverse transcriptase (RT) sequences. Statistical analysis was performed using SPSS v.20 software. Prevalence for resistance mutations was established according to the class of antiretroviral therapy.

**Results**: At least one resistance mutation was found in 166 (81.4%) of 204 adult patients included. For nucleoside reverse transcriptase inhibitors (NRTIs), the most frequent mutations were M184V (63.8%), L74V (17.6%), M41L (17.2%), T215Y (14.1%), D67N (12.2%), Y115F (7.5%) and K65R (2.5%). M184V was found along other resistance mutation to NRTIs in 91.2% of the cases. The most common resistance mutations for non-nucleoside reverse transcriptase inhibitors (NNRTIs) were K103N (34.3%), Y181C (13.4%), G190A (10.4%), P225H (8.0%), K101E (6.0%) and M230L (4.5%). Among protease inhibitors (PIs), the most commonly found mutations were I54L (11.0%), V82A (10.4%), M46I (10.3%) and I50L (4.5%). In 52 cases, the combination M184V+K103N was found; in 5 cases, the combination K103N+M184V+M46I was detected.

**Conclusions**: As high as 81.4% of the viral strains analyzed were resistant to one or more drugs. M184V and K103N mutations in the reverse transcriptase (RT) gene were the most common ones found in the Colombian patients who participated in this study. The high level of reduced susceptibility they induce to lamivudine and emtricitabine (M184V) and efavirenz (K103N) may impair the benefits of ART. Resistance mutations to PIs ranged from 4.5% to 11.0%.

# Sub article: P37: Switch from NNRTI plus FTC/TDF to E/C/F/TDF maintains HIV suppression and is well-tolerated

**DOI:** 10.7448/IAS.17.2.19177

**Published:** 2014-05-07

**Authors:** DeJesus Edwin, Flamm Jason, Pozniak Anton, Antela Antonio, Domingo Pere, Garner Will, Nguyen Thai

**Affiliations:** 1 Orlando Immunology Center, Infectious Diseases, Orlando, FL, USA; 2 Kaiser Permanente, Sacramento, Infectious Diseases, CA, USA; 3 Chelsea and Westminster Hospital, Infectious Diseases, London, UK; 4 Hospital Clinico Universitario, Infectious Diseases, Santiago de Compostela, Spain; 5 Hospital de la Santa Creu I Sant Pau, Infectious Diseases, Barcelona, Spain; 6 Biostatistics, Gilead Sciences, Foster City, CA, USA; 7 Medical Affairs, GIlead Sciences, Foster City, CA, USA

## Abstract

**Introduction**: Concerns with current and/or long-term side effects or dosing complexity of antiretroviral (ARV) regimen may prompt patients to request ARV switches. We report the Week (W) 48 results of a prospective, randomized, open-label, ongoing Phase 3b trial of a regimen switch to the single-tablet regimen (STR) elvitegravir/cobicistat/emtricitabine/tenofovir DF (E/C/F/TDF) from non-nucleoside reverse transcriptase inhibitor (NNRTI)+emtricitabine/tenofovir DF (FTC/TDF) regimens in virologically-suppressed HIV-1 subjects.

**Methods**: Subjects suppressed on NNRTI+FTC/TDF regimens for ≥six months were randomized (2:1) to switch to E/C/F/TDF or remain on their baseline NNRTI regimen (i.e., NNRTI). Eligibility criteria included CrCl ≥70 ml/min, no documented resistance to FTC and TDF, exposure to no more than two prior ARV regimens and no history of virologic failure. The primary endpoint was the proportion of subjects who maintained HIV-1 RNA <50 c/ml at W48 by FDA snapshot algorithm (12% non-inferiority margin).

**Results**: A total of 434 subjects (93% male, 22% non-white, 22% age ≥50 years) were randomized and treated (291 E/C/F/TDF; 143 NNRTI). At randomization, 78% of subjects were on an efavirenz (EFV)-based regimen (74% on STR EFV/FTC/TDF); median years since first ARV use was three; and 31% enrolled in the study due to concern with current or long-term side effects of their ARVs. Baseline characteristics were similar between the two groups. E/C/F/TDF was non-inferior to NNRTI regimens as 93% and 88% respectively maintained HIV-1 RNA <50 c/ml at W48 (difference 5.3%, 95% CI −0.5%, +12.0%). Virologic failure rates were 1% with no emergent resistance detected in either group. The safety and tolerability profiles of E/C/F/TDF were consistent with reports from previous studies. Grade 3 or 4 adverse events (AEs) were low and similar in both groups. AEs leading to discontinuation were low (2.1% E/C/F/TDF vs. 0.7% NNRTI). Median changes in CrCl (ml/min) at W48 were, as expected, −11.6 and −0.2 respectively. Small decreases from baseline in total, LDL and HDL cholesterols were experienced by those switching from EFV-based regimens. Decreases from baseline at W48 in rates of neuropsychiatric symptoms, e.g., vivid dreams (−15%, *p*<0.001), dizziness (−11%, p<0.001), anxiety (−9%, *p*=0.008) and insomnia (−10%, *p*=0.004), were reported after switching to E/C/F/TDF (HIV Symptom Index). HIV Treatment Satisfaction scores were higher for subjects who switched to E/C/F/TDF (*p*<0.001) (HIV Treatment Satisfaction Questionnaire).

**Conclusions**: Switching to E/C/F/TDF from NNRTI+FTC/TDF regimens was associated with high rates of virologic suppression, no resistance development and favourable tolerability with improved treatment satisfaction.

# Sub article: P38: Simplification of PI+RTV+FTC/TDF to E/C/F/TDF maintains HIV suppression and is well-tolerated

**DOI:** 10.7448/IAS.17.2.19178

**Published:** 2014-05-07

**Authors:** Shamblaw David, Cunningham Doug, Arribas Jose, Gathe Joseph, Ebrahimi Ramin, White Kirsten L, Nguyen Thai

**Affiliations:** 1 La Playa Medical Group and Clinical Research, Infectious Diseases/HIV, San Diego, CA, USA; 2 Pueblo Family Physicians, HIV, Phoenix, AZ, USA; 3 Hospital Universitario La Paz, Infectious Diseases, Madrid, Spain; 4 Baylor College of Medicine, Infectious Diseases, Houston, TX, USA; 5 Biostatistics, Gilead Sciences, Foster City, CA, USA; 6 Clinical Virology, Gilead Sciences, Foster City, CA, USA; 7 Medical Affairs HIV, Gilead Sciences, Foster City, CA, USA

## Abstract

**Introduction**: Antiretroviral (ARV) regimen simplification can improve treatment adherence and quality of life. We report the Week (W) 48 results of a prospective, randomized, open-label, ongoing Phase 3b trial of a regimen simplification to the single-tablet regimen elvitegravir/cobicistat/emtricitabine/tenofovir DF (E/C/F/TDF) from ritonavir-boosted protease inhibitor (PI+RTV) plus emtricitabine/tenofovir DF (FTC/TDF) regimens.

**Methods**: Virologically suppressed subjects on PI+RTV+FTC/TDF regimens for ≥six months were randomized (2:1) to switch to E/C/F/TDF or remain on their baseline PI regimen (i.e., PI). Eligibility criteria included CrCl ≥70 ml/min, no documented resistance to FTC and TDF, exposure to no more than two prior ARV regimens and no history of virologic failure. The primary endpoint was the proportion of subjects who maintained HIV-1 RNA <50 c/ml at W48 by FDA snapshot algorithm (12% non-inferiority margin). If non-inferiority was established, then superiority would be tested per a pre-specified sequential testing procedure.

**Results**: A total of 433 subjects (86% male, 19% non-white, 18% age ≥50 years) were randomized and treated (293 E/C/F/TDF; 140 PI). At randomization, RTV-boosted atazanavir (40%) and RTV-boosted darunavir (40%) were the most common PIs used; median years since first ARV use was 3; 19% were on their second ARV regimen. Baseline characteristics were similar between the two groups. At W48, 94% of subjects on E/C/F/TDF maintained HIV-RNA <50 c/ml compared to 87% on PI (difference 6.7%, 95% CI +0.4% to +13.7%; *p*=0.025). Rates of virologic failure were low (0.7% E/C/F/TDF vs. 1.4% PI) with no emergent resistance in either group. The safety and tolerability profiles of E/C/F/TDF were consistent with those reported in previous studies. Grade 3 or 4 adverse events (AEs) were low and similar in both groups. AEs leading to drug discontinuation were low, 2.0% vs. 2.9% respectively. At W48, median changes in CrCl (ml/min) were −7.5 and 0.4, respectively, with no cases of proximal renal tubulopathy in either group. There was a larger decrease from baseline in fasting triglycerides for E/C/F/TDF compared to PI (median: −16 vs. +3 mg/dl; *p*=0.001) and no change in other lipid parameters.

**Conclusions**: Switching to E/C/F/TDF compared to continuing PI+RTV+FTC/TDF resulted in significantly higher rates of virologic suppression without emergence of resistance. E/C/F/TDF was well-tolerated with a favourable safety profile. Switching to E/C/F/TDF from a multiple-tablet, PI-based regimen may be an option for patients wishing to simplify their ARV therapy.

# Sub article: P39: Effectiveness of tenofovir/3(F)TC plus NNRTI compared to zidovudine/3TC plus NNRTI first-line regimens in Argentina

**DOI:** 10.7448/IAS.17.2.19179

**Published:** 2014-05-07

**Authors:** Bissio Emiliano, Balleri Cynthia, Falistocco Carlos

**Affiliations:** Direction of AIDS, Ministry of Health, Buenos Aires, Argentina

## Abstract

**Introduction**: In the last years, in concordance with WHO recommendations, the Direction of Aids in Argentina has been working to maximize universal access to HIV care. In addition, a program aiming at improving quality of care is also being implemented. As part of this program, use of simpler, easy-to-use and less toxic ARV formulations is encouraged. Latest guidelines launched by Argentina's Ministry of Health recommend NNRTI-based regimens as first-line therapy, associated to tenofovir/emtricitabine, tenofovir/lamivudine or abacavir/lamivudine, whilst zidovudine/lamivudine remains also as an option for use as first line. The aim of this study was to compare effectiveness of tenofovir/3(F)TC plus NNRTI with zidovudine/lamivudine plus NNRTI first-line regimens at population level, measured as the proportion of persons with suppressed viremia.

**Methods**: Cross-sectional study. The proportion of patients on stable antiretroviral treatment (>six months) served by Argentina's Direction of Aids (approximately 70% of all HIV-infected persons) who were virologically suppressed was determined by the end of 2013. The patients were divided into two groups; those on first-line NNRTI-based tenofovir/F(3)TC regimens and those on NNRTI-based AZT/3TC. Virological suppression was defined as plasma viral load ≤50 copies/ml. NNRTIs used were efavirenz or nevirapine.

**Results**: Out of approximately 35,000 persons on antiretrovirals delivered by the Direction of Aids during 2013, viral load data were retrieved for 12,037; mean(SD) age was 41(11.2) years, 40.38% were female. Of these, 6281 (52.2%) persons were on first-line NNRTI-based regimens. Within this population, a total of 1055 persons were on tenofovir/3(F)TC regimens for more than six months and 2749 on AZT/3TC. The suppression rate among people in the tenofovir/3(F)TC group was 75.1% (792/1055), versus 71.2% for the AZT/3TC group (1958/2749); *p*=0.02.

**Conclusions**: In Argentina, first-line NNRTI-based regimens containing tenofovir/3(F)TC seem to be more effective than their zidovudine/3TC counterparts when evaluated at a population level. Tolerability and easiness to take may contribute to this phenomenon. These data support WHO recommendations for first-line regimens containing tenofovir/3TC or tenofovir/FTC as preferred. However, due to the cross-sectional and observational design, potential biases must be considered.
